# Prenatal Exposure to Size-Fractionated Particulate Matter and Fetal Growth Trajectories: Mediation by Maternal Glucose Metabolism in a Birth Cohort

**DOI:** 10.3390/toxics14080709

**Published:** 2026-08-12

**Authors:** Qian Chen, Bijun Shi, Yu Liu, Xin Wang, Li Cai, Qiliang Cui, Xiaohua Tan, Yujing Chen

**Affiliations:** 1 Department of Child Healthcare, The Third Affiliated Hospital, Guangzhou Medical University, Guangzhou 510150, China; 2 Guangdong Provincial Key Laboratory of Major Obstetric Diseases, Guangdong Provincial Clinical Research Center for Obstetrics and Gynecology, Guangdong-Hong Kong-Macao Greater Bay Area Higher Education Joint Laboratory of Maternal-Fetal Medicine, Guangzhou 510150, China; 3 Department of Maternal and Child Health, School of Public Health, Sun Yat-sen University, Guangzhou 510080, China; 4 Key Laboratory of Brain, Cognition and Education Sciences, Ministry of Education, Institute for Brain Research and Rehabilitation, Guangdong Key Laboratory of Mental Health and Cognitive Science, South China Normal University, Guangzhou 510631, China

**Keywords:** PM_2.5_, PM_1_, glucose metabolism, pregnancy, insulin resistance, fetal growth trajectory

## Abstract

The metabolic mechanisms linking prenatal particulate matter (PM) to fetal growth remain unclear. A prospective cohort of 616 mother–fetus pairs in Guangzhou had ultrasound-based fetal growth parameters repeatedly measured from mid to late pregnancy. Fetal head circumference (HC), abdominal circumference (AC), femur length (FL), and biparietal diameter (BPD) were standardized as z-scores. Estimated fetal weight (EFW) trajectories were classified as average, slow, and rapid growth. Residential PM_1_, PM_2.5_, and PM_10_ were assessed with 1 km spatiotemporal models. Multinomial logistic regression analyzed PM-EFW trajectory associations. Generalized estimating equations evaluated PM–fetal growth z-score relationships. Maternal plasma glucose and insulin levels at mid-pregnancy were examined as intermediate factors. Each 5 μg/m^3^ increase in PM was associated with higher odds of rapid EFW growth versus average growth (OR _PM1_ = 1.56, 95% CI: 1.03, 2.37; OR _PM2.5_ = 1.44, 95% CI: 1.10, 1.88; OR _PM10_ = 1.28, 95% CI: 1.05, 1.56). Positive associations were also observed for BPD, HC, and AC z-scores, with PM_1_ showing the numerically larger effect estimates. Maternal fasting plasma glucose and insulin resistance may partly explain the observed associations of PM exposure with rapid EFW growth and fetal growth parameters, with estimated proportions of 5.0–29.4%. This study highlights the potential risk of accelerated fetal growth associated with maternal PM exposures, and maternal glucose metabolism may represent a plausible pathway.

## 1. Introduction

Air pollution is a leading environmental threat impacting health outcomes throughout the life course [1]. Developing fetuses are at higher health risks [2], as ambient particulate matter (PM) can adsorb toxic agents and cross the placenta [3], potentially disrupting the intrauterine environment and fetal development through multiple pathophysiological mechanisms [4,5,6]. Both insufficient and excessive fetal growth represent departures from optimal intrauterine development. Although epidemiological research has traditionally focused on fetal growth restriction, fetal overgrowth is also an important health concern [7,8]. It has been associated with adverse maternal and neonatal outcomes, including labor complications [8,9], as well as increased risks of obesity and cardiometabolic disorders later in life [10,11].

Recent systematic reviews of epidemiological studies have linked prenatal exposure to PM with aerodynamic equivalent diameter ≤ 2.5 μm (PM_2.5_) to birth weight outcomes, including both decreased term birth weight and macrosomic birth [12,13]. However, fetal growth patterns may not be fully characterized by birth weight alone, and the size-specific effects of PM on longitudinal fetal growth remain understudied. Particle size may be biologically relevant, as smaller particles have higher surface-area-to-mass ratios and surface reactivity, which may facilitate the adsorption and transport of potentially toxic constituents [14]. Current investigations into prenatal size-fractionated PM exposure and intrauterine growth patterns remain limited and yield inconsistent findings. For example, several cohort studies from China [15,16] and the USA [17] indicate the growth-restricting effects of PM, reporting associations between early pregnancy PM_2.5_ and reduced ultrasound-based fetal growth parameters. In contrast, cohorts from the Netherlands [18] and Spain [19] observed that PM with aerodynamic equivalent diameter ≤ 10 μm (PM_10_) and PM_2.5_ were associated with increased fetal growth during the second and third trimesters. Another study in California further linked PM_2.5_ to fast growth rates from the third trimester to age 3 months [20]. These mixed findings may reflect bidirectional effects of air pollutants on fetal growth [21], and PM-induced growth delays might be offset by accelerated growth in late pregnancy [15,22]. Therefore, longitudinal assessment of fetal growth in utero may provide additional insight into the heterogeneity of environmentally related growth profiles. Additionally, smaller particles may differ from larger particles in their physicochemical properties and potential health effects [23,24]. Recent studies have also highlighted the contribution of submicron particles (PM_1_, aerodynamic diameter ≤ 1 μm) to urban air pollution [25]. However, the risk of PM_1_ on fetal growth has received limited attention [26,27], partly because ground-level PM_1_ monitoring data for population-based studies remain scarce.

PM may affect fetal growth through interrelated pathways, including placental inflammation, oxidative stress, endothelial dysfunction, and maternal endocrine and metabolic disturbances [4,5,6,28]. Among these, altered maternal glucose metabolism may be particularly relevant to fetal growth, as elevated maternal glucose levels may increase transplacental glucose transfer and promote fetal tissue accretion [29,30]. PM-induced inflammation and oxidative stress may, in turn, contribute to maternal dysglycemia, potentially altering the metabolic environment and nutrient supply that regulate fetal growth [31]. Our previous study has suggested that PM_2.5_ was associated with increased maternal gestational and postpartum fasting glucose levels [32]. Recent studies also indicated that gestational diabetes mellitus (GDM) may modify the PM_2.5_-associated fetal growth velocity [33], and that PM exposure may increase birth weight by elevating maternal fasting glucose levels [34]. Nevertheless, whether maternal glucose metabolism mediates the association between prenatal PM exposure and longitudinal fetal growth in utero remains unclear.

Using high-resolution modeling data on PM exposure, this prospective birth cohort aims to fill research gaps by: (1) investigating the longitudinal association between prenatal exposure to PM_1_, PM_2.5_, and PM_10_ and fetal growth trajectories in utero, and (2) examining the potential mediating role of maternal glucose metabolism in these associations. We hypothesized that PM exposure would be associated with latent class-based fetal growth trajectories, with effects varying by particle sizes, and that maternal glucose biomarkers may act as mediators.

## 2. Materials and Methods

### 2.1. Study Population

**Figure 1:**
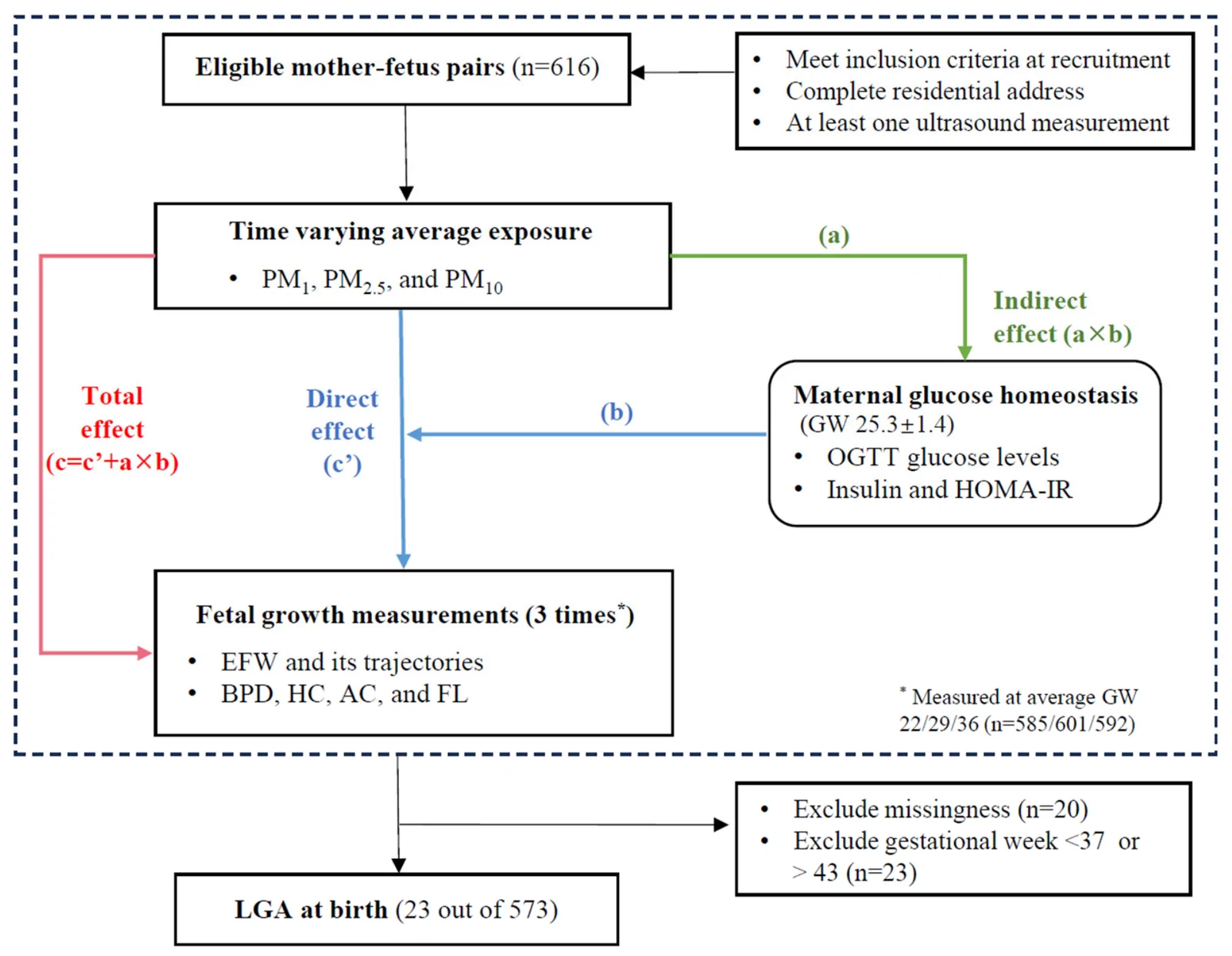
Flow chart of participant inclusion and mediation framework for fetal growth in utero. Abbreviations: PM_1_, particulate matter with aerodynamic diameters ≤ 1 μm; PM_2.5_, particulate matter with aerodynamic diameters ≤ 2.5 μm; PM_10_, particulate matter with aerodynamic diameters ≤ 10 μm; BPD, biparietal diameter; HC, head circumference; AC, abdominal circumference; FL, femur length; EFW, estimated fetal weight. FPG, fasting plasma glucose; OGTT, oral glucose tolerance test; HOMA-IR, homeostatic model assessment for insulin resistance; GW, gestational week; LGA, large for gestational age. Inclusion criteria at recruitment were: (1) 20–45 years of age, (2) without pregestational diabetes mellitus (diabetes diagnosed before the current pregnancy), cardiovascular disease, thyroid disease, hematopathy, polycystic ovary syndrome, pregnancy infection, or mental disorder, and (3) singleton pregnancy. Women who developed GDM during the current pregnancy were retained because gestational glucose metabolism was a primary pathway of interest. Mediation analysis (in the dashed box) shows that the total effect (c) of PM exposure was decomposed into the indirect effect (a × b) and direct effect (c′). The effects and their 95% confidence intervals were computed using percentile bootstrap (2000 resamples). The proportion mediated was calculated within each bootstrap sample as the indirect effect divided by the total effect, and the mean of the bootstrap-specific proportions was reported. Mediation model: a, coefficient of exposure where the mediator was the outcome; b, coefficient of mediator where exposure and mediator were included as predictors for the outcome. c′, coefficient of exposure where exposure and mediator were included as predictors for the outcome.

This birth cohort study in Guangzhou, China (NCT03023293) recruited pregnant women between 20 and 28 weeks of gestation at the Yuexiu District Maternal and Child Health Hospital between 2017 and 2018. Inclusion criteria were: (1) 20–45 years of age, (2) without pregestational diabetes mellitus, cardiovascular disease, thyroid disease, hematopathy, polycystic ovary syndrome, pregnancy infection, or mental disorder, and (3) singleton pregnancy. This final study sample consisted of 616 mother–fetus pairs who had at least one ultrasound measurement for fetal growth and provided a complete residential address during pregnancy (Figure 1). The institutional review boards of Sun Yat-sen University approved the study protocol, and written informed consent was obtained from all participants (registration number: NCT03023293; Approval No.: SYSU-PHME [2016] No. 014, Date: 28 December 2016).

### 2.2. Fetal Growth Measurements

Routine ultrasound examinations were conducted by sonographers during the second and third trimesters. GA was determined according to the last menstrual period (LMP) and verified by an early ultrasound examination in the first trimester. About 90.26% of women underwent three ultrasound scans at average GAs of 22, 29, and 36 weeks. Fetal growth parameters include head circumference (HC), abdominal circumference (AC), femur length (FL), and biparietal diameter (BPD). Estimated fetal weight (EFW) was calculated using the Hadlock formula, incorporating AC, HC, and FL [35]. These parameters were then converted to GA- and sex-adjusted z-scores using Generalized Additive Models for Location, Scale, and Shape [36]. Fetal overgrowth was defined as z-score > 1.88 (>97th centile) [22]. Newborns were classified as large for gestational age (LGA, 90th percentile) and small for gestational age (SGA, <10th percentile) based on Chinese birth weight standards, respectively [37].

### 2.3. Exposure Assessment

PM mass concentrations were retrieved from the China High Air Pollutants (CHAP) dataset [38,39,40,41]. These estimates were generated using a Space–Time Extra-Trees model that integrates satellite remote sensing, ground-based observations, meteorological variables, land-use information, and other auxiliary factors. The model captures complex nonlinear relationships and generates spatially continuous estimates, including for locations or days with missing satellite observations. Ground measurements used for model development and validation were obtained from nationwide monitoring networks, including the China National Environmental Monitoring Centre network and the China Atmosphere Watch Network. The model provides daily PM estimates at a 1 km × 1 km spatial resolution with high predictive accuracy. At the national scale, the cross-validation coefficients of determination (CV-R^2^) and root-mean-square errors (RMSE) were 0.83 and 9.50 μg/m^3^ for PM_1_, 0.92 and 10.76 μg/m^3^ for PM_2.5_, and 0.90 and 21.12 μg/m^3^ for PM_10_, respectively. The study participants were located in the Pearl River Delta, which has a dense network of ground monitoring stations (Figure S1). Previous CHAP studies also reported good regional predictive accuracy for PM_2.5_ (CV-R^2^ = 0.86) and PM_10_ (CV-R^2^ = 0.84), while station-level validation maps indicated similarly satisfactory predictive performance for PM_1_ in Guangdong [39,40,41].

Residential exposure was assigned based on the maternal residential address at enrollment. Residential addresses were geocoded and linked to the corresponding 1 km CHAP grid. To capture exposure during different stages of fetal development, daily PM concentrations were averaged over three ultrasound-specific exposure windows (from conception to each ultrasound examination) and over the entire pregnancy (from conception to delivery) to derive mean prenatal exposures.

### 2.4. Glucose Metabolism

Pregnant women underwent a 75 g oral glucose tolerance test (OGTT) at 24–28 weeks of gestation, the recommended period for screening gestational diabetes [42]. Plasma glucose levels (mmol/L), including fasting plasma glucose (FPG), 1 h glucose, and 2 h glucose, were measured with a clinical chemistry analyzer (ARCHITECT i2000SR; Abbott, Abbott Park, IL, USA) using the glucose oxidase method. Fasting insulin concentration was measured using an enzyme-linked immunosorbent assay (10-1113-01; Mercodia, Uppsala, Sweden). The homeostasis model assessment of insulin resistance (HOMA-IR) index was calculated by the formula: fasting insulin (μU/mL) × FPG (mmol/L)/22.5.

### 2.5. Covariates

Maternal sociodemographic characteristics were collected during the baseline interview, including maternal age, pre-pregnancy BMI (kg/m^2^), maternal education level, employment status, monthly household income, and parity. Maternal passive smoking was defined as exposure to secondhand smoke for more than 15 min per day for at least 1 day per week during pregnancy (yes or no). Weekly physical activity (METs·h/w) and daily dietary energy intake (kcal/day) were investigated using standardized questionnaires. Season of conception was estimated based on the month of LMP (summer–autumn: June to November; winter–spring: December to May). Fetal sex was obtained from medical records.

The daily average temperature was sourced from the National Oceanic and Atmospheric Administration. Daily nitrogen dioxide (NO_2_) and maximum 8 h average ozone (O_3_) concentrations at 10 km spatial resolution were derived from CHAP datasets. We estimated the average temperature, NO_2_, and O_3_ during pregnancy for each participant.

### 2.6. Statistical Analysis

Descriptive statistics were used to summarize participants’ characteristics, with continuous variables expressed as means and standard deviations (SDs) and categorical variables as frequencies and percentages.

Growth trajectories of EFW were modeled using the latent class mixed model (LCMM), assuming heterogeneity in the population, with latent classes defined by distinct mean profiles of longitudinal trend [43]. The model included a random-effects structure with linear and quadratic functions for class-specific and individual random effects. An optimal model with three latent classes was determined based on information criteria, as shown in Figure S2. Individuals were assigned to the trajectory with the highest posterior probabilities (Table S1). The multinomial logistic regression model was employed to assess the association between PM during pregnancy and FEW trajectory classes.

Longitudinal association between repeated measures of PM and continuous fetal growth z-scores was examined using the generalized estimating equation (GEE). An independence correlation matrix and robust variance estimators were employed based on the quasi-information criterion. The main analytical model was adjusted for a minimally sufficient set of covariates identified using a directed acyclic graph (Figure S3) and literature [15,17,34,44], including maternal age, maternal education, employment status, household income, physical activity, pre-pregnancy BMI, parity, fetal sex, season of conception, and average temperature during pregnancy. Restricted cubic splines with three knots were used to explore potential nonlinear associations between PM and fetal growth.

Associations between glucose biomarkers and fetal growth were examined, and their potential mediating roles were evaluated using the product method [45]. As depicted in Figure 1, the estimated total effect (c) of PM exposure in relation to fetal growth outcomes was decomposed into the indirect effect (a × b) and direct effect (c′). Effects and their 95% confidence intervals were computed using percentile bootstrap (2000 replications). For specific fetal growth trajectories significantly associated with PM, mediation analysis was conducted based on whole-pregnancy PM exposure to capture overall cumulative exposure. Because the trajectory classification incorporated all available ultrasound measurements from mid-pregnancy onward, strict temporal separation among PM exposure, glucose assessment, and fetal growth was not fully achieved. These analyses were interpreted as evaluating a potential pregnancy-level metabolic pathway rather than establishing causal mediation. GEE-based mediation analyses were additionally conducted for repeatedly measured fetal growth parameters using PM exposure windows estimated from conception to each ultrasound examination [46,47]. The primary mediation models were adjusted for the same prespecified covariates as the corresponding PM–fetal growth models.

Several supplementary and sensitivity analyses were performed to: (1) examine pooled associations across five imputed datasets for covariates. Given the low missing rate and comparable results across imputed datasets in Table S2, we randomly selected one complete dataset for the main analysis; (2) additionally adjust for potential determinants of outcomes (dietary energy intake, passive smoking, NO_2_, and O_3_) in both the PM–fetal growth and mediation models; (3) analyze longitudinal association between fetal overgrowth (z-score > 1.88) and PM exposures; (4) additionally evaluate associations with birth weight, SGA, and LGA to test the clinical relevance of growth trajectories and analyze association between PM and these weight outcomes at birth. Due to the low rates of fetal overgrowth and LGA, Firth’s correction was applied to the penalized likelihood in regression models [48]; (5) re-run the GEE-based mediation analysis following a stricter temporal sequence, using PM exposure assessed from conception to the first ultrasound examination at approximately gestational week 22, glucose biomarkers measured at approximately gestational week 25, and fetal growth outcomes measured thereafter.

All analyses were conducted using R 4.2.0 (R Core Team, 2022), with R packages including “lcmm” (version 2.2.1), “nnet” (version 7.3.20), “geepack” (version 1.3.12), and “mediation” (version 4.5.0). A *p*-value < 0.05 for a two-sided test was considered statistically significant.

## 3. Results

### 3.1. General Characteristics of the Study Population

As shown in Table 1, a total of 616 mother–child pairs were included in this study, with a mean maternal age of 30.54 ± 4.87 years. Among the fetuses, 48.54% (299/616) were male and 4.01% (23/573) were LGA at birth. During the entire pregnancy, the mean concentrations of PM_1_, PM_2.5_ and PM_10_ were 17.67 ± 2.40 μg/m^3^, 34.47 ± 3.67 μg/m^3^, and 57.25 ± 5.21 μg/m^3^, respectively. The pairwise correlations between the PM and environmental factors are presented in Figure S4.

Table S3 shows the cumulative PM exposures up to three ultrasound scan dates, along with the corresponding original scales and z-scores of fetal growth parameters. The mean EFW was 522.99 g, 1441.13 g, and 2764.93 g at around gestational weeks 22, 29, and 36.

### 3.2. Association Between PM and EFW Trajectories and Fetal Growth Parameters

**Figure 2:**
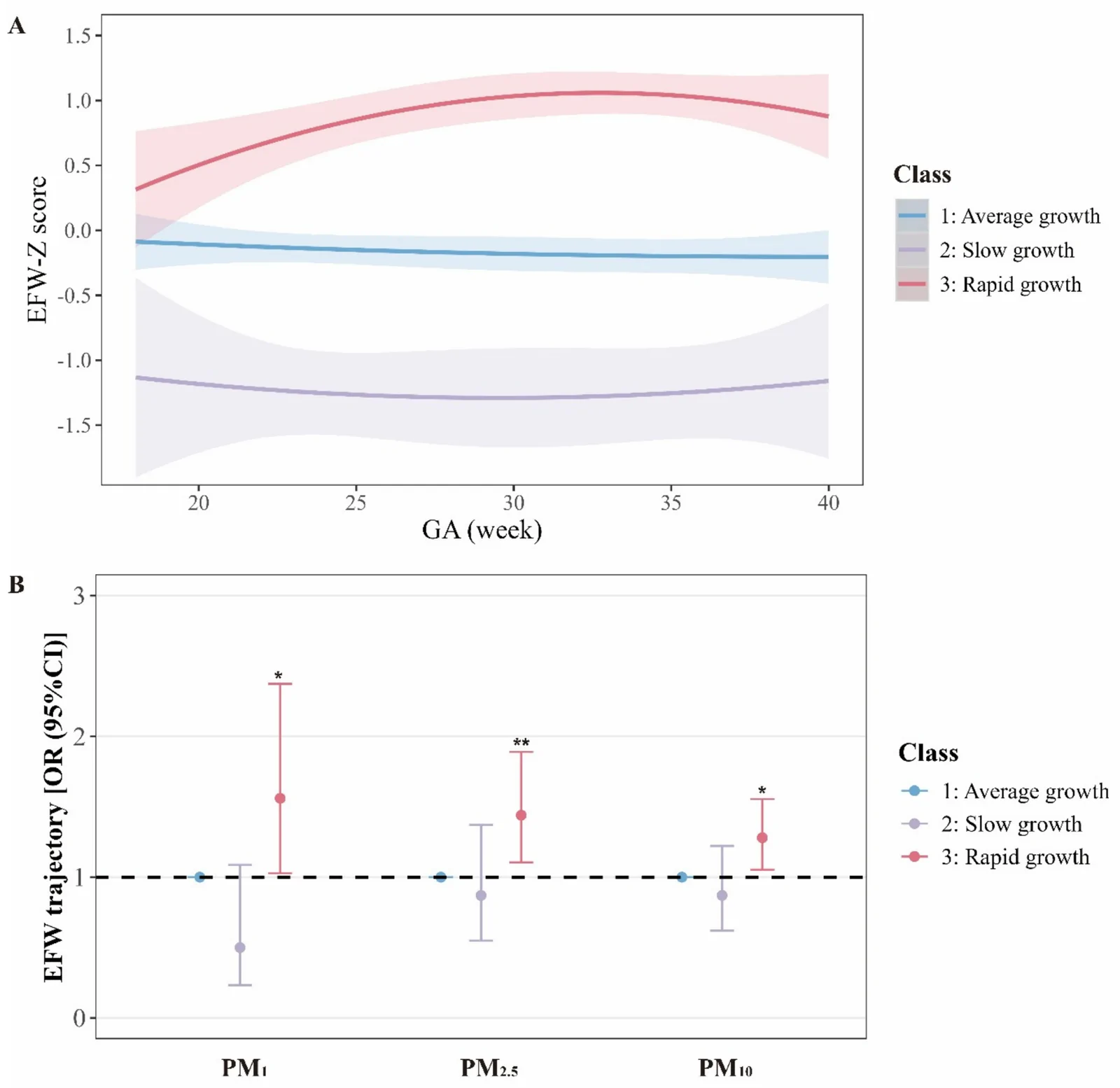
The growth trajectory classes of estimated fetal weight (EFW) in utero in association with maternal exposure to PM (per 5 μg/m^3^) in 616 mother–fetus pairs. (**A**) Growth trajectories of EFW z-score based on the latent class mixed models. (**B**) Association between PM exposure and EFW trajectories. The average-growth trajectory was the reference category, with its OR fixed at 1.0. The horizontal dashed line indicates the null value (OR = 1.0). Abbreviations: EFW, estimated fetal weight; PM_1_, particulate matter with aerodynamic diameters ≤ 1 μm; PM_2.5_, particulate matter with aerodynamic diameters ≤ 2.5 μm; PM_10_, particulate matter with aerodynamic diameters ≤ 10 μm. Class 1: average growth (*n* = 420); class 2: slow growth (*n* = 47); class 3: rapid growth (*n* = 149). They are labeled according to the stable z-score near the null (reference group), persistently low z-score, and high-rising trends of z-score, respectively. Effect estimates are reported per 5-μg/m^3^ increase in exposure. The model was adjusted for maternal age, maternal employment status, household income, maternal education, physical activity, pre-pregnancy BMI, parity, fetal sex, season of conception, and average temperature during pregnancy. * *p* < 0.05, ** *p* < 0.01.

Figure 2 illustrates the EFW growth trajectories for three classes: average (reference), slow, and rapid growth. These trajectories are characterized by stable null, persistent low, and high-rising z-scores during the second and third trimesters, respectively. In the adjusted model, per 5 μg/m^3^ increase in PM_1_, PM_2.5_, and PM_10_ during the pregnancy was associated with an odds ratio (OR) of 1.56 (95% CI: 1.03, 2.37), 1.44 (95% CI: 1.10, 1.88), and 1.28 (95% CI: 1.05, 1.56) for rapid growth of EFW, respectively. No association was observed for slow growth trajectory.

In the GEE-based model (Table S4), PM exposure was associated with increased BPD, HC, AC, and EFW, except for FL. PM_1_ tended to show numerically larger coefficients for these fetal growth parameters, with *β* values ranging from 0.064 to 0.108 per 5-μg/m^3^ increase in exposure. Figures S5 and S6 suggest linear relationships between PM exposure and fetal growth outcomes.

### 3.3. Mediation Analyses of Glucose Biomarkers

Maternal FPG, insulin, and HOMA-IR showed significantly positive associations with fetal growth outcomes (Tables S5 and S6). In Table 2, insulin and HOMA-IR may partly explain the associations of PM_1_ and PM_10_ with the rapid EFW growth trajectory. The corresponding mediation proportions were 16.0% and 21.9% for insulin and 15.7% and 22.2% for HOMA-IR, respectively. In addition, FPG may partly explain the associations of PM_2.5_ and PM_10_ with the rapid growth trajectory, accounting for 10.3% and 12.5% of the observed associations.

For continuous fetal growth parameters (Table 3), maternal FPG, insulin, and HOMA-IR also showed potential indirect effects in the associations between PM exposure and EFW, BPD, HC, and AC z-scores. The estimated mediation proportions ranged from 5.0% to 29.4% across pollutant–glucose biomarker–outcome combinations. For PM_10_, relatively larger mediation proportions were observed for insulin and HOMA-IR in several associations (14.0–29.4%). Overall, these glucose biomarkers may explain a modest proportion of the observed PM–fetal growth associations, leaving the majority unexplained.

### 3.4. Supplementary and Sensitivity Results

The associations of PM and fetal growth were robust when adjusting for dietary energy intake, passive smoking, NO_2_, and O_3_ (Table S7) and using the multiple-imputed datasets (Table S8). We also observed suggestive associations between PM exposures and overgrowth risks for head development (BPD and HC) and AC (*p* < 0.05 or 0.10, Table S9). Slow and rapid EFW growth trajectories were significantly associated with decreased and increased birth weight (Table S10), while PM exposure (PM_1_ and PM_2.5_) only showed a positive association with LGA (Figure S7). The potential indirect effects through maternal glucose biomarkers also remained generally consistent after additional adjustment for other lifestyle factors and co-pollutants. For the rapid EFW growth trajectory, the estimated proportions ranged from 7.6% to 11.5% for PM_10_, whereas those for repeated EFW z-scores ranged from 7.2% to 10.5% across PM size fractions (Table S11). Under a stricter temporal sequence, potential indirect effects remained evident for selected associations of PM exposure with EFW, BPD, HC, and AC z-scores, with estimated proportions ranging from 6.9% to 37.3% (Table S12).

## 4. Discussion

This prospective cohort study demonstrated a consistent association between size-specific PM exposure and the rising growth trajectory of EFW during pregnancy. We also observed longitudinally positive associations between PM and z-scores of fetal BPD, HC and AC. Maternal fasting glucose biomarkers may partly explain the PM–fetal growth association. This study bridges existing knowledge gaps by examining whether size-fractionated PM-related hyperglycemia may disrupt dynamic fetal growth in utero.

Previous studies on PM exposure and fetal growth have reported mixed results, with some studies aligning with our findings [18,19,22] and others indicating growth-restricting effects of PM [15,16,17,28,49,50]. These inconsistencies may stem in part from heterogeneities in exposure and outcome assessments. Our study utilized high spatiotemporal resolution data to estimate residential PM exposure before outcome measurements rather than relying on methods of inverse distance weighting [22,33] or estimating emissions during the calendar year instead of pregnancy [49]. Meanwhile, while many studies have assessed the raw values of fetal outcomes [17,28,50,51], our study examined the standardized z-scores to account for the effect of GA. The positive associations we observed may reflect accelerated fetal growth in mid and late pregnancy, supporting previously reported excessive catch-up growth associated with air pollutants during birth or in postnatal periods [15,20,52]. A recent systematic review has highlighted the fetal overgrowth risks (measured by LGA and macrosomia) related to PM_2.5_ in 21 cohort studies [13]. A large Chinese population-based study of over 3 million newborns also suggested a PM_2.5_-associated increased risk of macrosomia [53]. In utero, the Spanish INMA birth cohort observed that cumulative PM_2.5_ exposure (per 5 μg/m^3^) during gestational weeks 29–33 was associated with a 0.42% increased EFW in late pregnancy [19]. A study in the Netherlands also reported that maternal PM_10_ exposure in the higher quartile levels across pregnancy was associated with an increased EFW during weeks 20–24 [18]. Our study was in line with these studies and provided new insights by capturing the dynamic features and distinct profiles of longitudinal fetal growth trends in utero. We also found the rapid EFW trajectory and PM were significantly associated with LGA risks at birth, underscoring the potential clinical relevance of observed associations in utero.

This study also examined the relationship between PM of different aerodynamic sizes and fetal BPD, HC, and AC, consistently finding a positive association. The smaller PM may have a larger surface area to carry toxins, and they can penetrate deeper into the gas-exchange region of the lungs and enter the systemic circulation [14]. To our knowledge, only two studies from a cohort in Beijing have examined PM_1_ in association with reduced z-scores of fetal parameters (i.e., EFW, AC, HC, and FL) [26,27]. However, positive associations of PM_2.5_ and PM_1_ with infant weight for length were found in the postnatal follow-up study of this cohort, with an effect size of 0.105 and 0.063 for PM_1_ and PM_2.5_ (per 10 μg/m^3^ increase) [54]. In our study, the consistent associations of PM across different particle sizes may reflect shared toxic components and common emission sources such as traffic exhaust. PM_1_ appeared to have numerically larger point estimates when exposure increments were held constant. Given that PM fractions are highly correlated and compositionally nested, the observed differences should be interpreted as a descriptive tendency rather than as definitive evidence of differential toxicity. Additionally, we found that PM was strongly associated with head development, with sensitivity analysis indicating increased risks of HC and BPD overgrowth (>97th centiles), despite the low rates. Head overgrowth in late pregnancy may result in adverse perinatal outcomes, such as shoulder dystocia, perinatal asphyxia, and cesarean section [9,55]. Future studies should further dissect the size-specific toxicity of PM to better understand its effects on fetal overgrowth risk.

Maternal glucose metabolism may present one plausible pathway linking PM exposure and the risk of fetal overgrowth. PM exposure has been shown to induce gestational hyperglycemia, and maternal diabetes can lead to larger fetal size [56,57]. It is plausible that excess maternal glucose can cross the placenta and stimulate fetal insulin secretion, thereby promoting fat deposition and abnormal weight gain, contributing to the onset of fetal overgrowth [58]. We are only aware of one study exploring the plausible mediating roles of maternal glucose levels on birth weight, which suggested positive indirect effects of PM_2.5_ and PM_10_ by increasing fasting glucose levels [34]. In that study, maternal fasting glucose mediated 63.6% (β = 8.2, 95% CI: 4.9, 12.1 g per 10 μg/m^3^) of the total effect of PM_2.5_ in the second trimester, with its direct effect (β = 4.7, 95% CI: −13.2, 19.2) trending toward null. Our previous study also indicated that PM_2.5_ specifically interfered with maternal fasting glucose [32], and this study further suggested that insulin resistance may partly contribute to the association with increased fetal growth in utero. Insulin resistance may also reflect lipid metabolic disorders, such as elevated triglyceride/high-density lipoprotein cholesterol (TG/HDL-C) ratios [59]. Higher maternal TG/HDL-C ratios have been related to both PM_2.5_ exposure and LGA infants [60]. In addition, the associations of smaller PM that were not fully explained by glucose biomarkers may be attributable to circulating adipokines, such as leptin and adiponectin. Maternal plasma leptin levels peak during late pregnancy and are susceptible to air pollution [61,62], potentially leading to metabolic changes and excessive fetal weight gain [63]. However, mid-pregnancy glucose biomarkers used in this study may partly reflect metabolic adaptations of the developing fetoplacental unit through placental endocrine signaling [64]. These potentially bidirectional interactions warrant confirmation in future studies with repeated metabolic assessments and clearer temporal ordering.

Nevertheless, maternal glucose biomarkers explained only a modest proportion of the observed associations in our study, suggesting that additional biological pathways may be involved, including placental dysfunction, oxidative stress and inflammation, endocrine disruption, and epigenetic alterations [4,6,28,65]. PM-induced oxidative stress and systemic inflammation may have contributed to altered maternal vascular and placental function, potentially affecting the intrauterine environment and the regulation of fetal growth [4,65]. Experimental evidence also suggests that particulate air pollution can modify placental morphology and potentially affect placental exchange function [6]. In addition, prenatal PM_2.5_ exposure has been associated with altered placental DNA methylation and fetal growth parameters, with methylation changes in selected fetal growth-related genes potentially contributing to these associations [28]. These pathways may act independently or interact with maternal glucose and lipid dysregulation, although their specific roles in fetal overgrowth warrant further investigation.

Unlike what is typically assumed, our study highlights the accelerated fetal growth risk induced by PM exposures. Monitoring maternal glucose metabolism may benefit maternal health and help identify pregnancies at increased risk of excessive fetal growth. The findings remain robust after adjusting for multiple lifestyle factors and other air pollutants (i.e., NO_2_ and O_3_). However, several limitations are acknowledged. First, exposure misclassification cannot be excluded. Residential mobility and occupational PM exposure during pregnancy were not collected, which may have introduced non-differential exposure misclassification and attenuated the observed associations. Second, maternal clinical information was not comprehensively characterized. We applied prespecified exclusion criteria for major maternal diseases at recruitment and collected data on gestational glucose metabolism, glucose-lowering treatment, and selected pregnancy complications. Glucose-lowering treatment and severe pregnancy complications were uncommon, with only three women reporting insulin use, none reporting oral glucose-lowering medications, one diagnosed with intrahepatic cholestasis of pregnancy, and seven diagnosed with preeclampsia. Nevertheless, other treatment use, selected laboratory factors such as hemoglobin and inflammatory biomarkers, and fetal growth and other perinatal outcomes in previous pregnancies were not comprehensively evaluated. Although the low frequency of glucose-lowering treatment and severe pregnancy complications reduces concern about substantial bias, residual confounding due to incompletely measured maternal and clinical factors cannot be excluded. Third, the temporal overlap among PM exposure, mid-pregnancy glucose assessment, and fetal growth measurements limits the causal interpretation of the mediation findings. Maternal glucose metabolism was assessed only at mid-pregnancy, a sensitive window for detecting gestational glucose intolerance [42]. Because approximately 30% of ultrasound measurements preceded or occurred close to the OGTT, reverse causality or bidirectional maternal–placental–fetal metabolic feedback cannot be excluded. Nevertheless, the trajectory analysis captured overall growth patterns across mid- to late pregnancy, with class differences becoming more pronounced during late gestation, and our sensitivity analyses with stricter temporal ordering yielded comparable results. Fourth, we only collected three routine ultrasound scans for fetal growth from mid to late pregnancy following Chinese guidelines [66]. Nonetheless, this approach allowed us to examine the longitudinal association with consistent growth parameters during the periods when most of the constitutional variation in growth occurs. Further studies should consider fetal growth monitoring data at more time points to better model fetal growth trajectories from early pregnancy. Fifth, other potential pathways linking PM to fetal growth remain unexplored. Future longitudinal studies incorporating repeated inflammatory and metabolic assessments from pregnancy through postpartum, together with postnatal offspring follow-up, are needed to clarify additional biological pathways and longer-term maternal and child health implications. Finally, this cohort was conducted in a central district of Guangzhou, where PM exposures are generally higher than in Western countries [17,19,49] but lower than in northern cities in China [22,26,27]. Recent declines in pollutant levels and changes in source profiles may limit direct extrapolation of our findings, although the exposure range remains relevant to populations with comparable or higher pollution levels in China. Future multi-center cohorts with larger sample sizes should be conducted to evaluate temporal and geographic heterogeneity and confirm our findings.

## 5. Conclusions

This prospective cohort study consistently observed positive associations between prenatal exposure to size-specific PM and rapid growth trajectory of EFW, with maternal fasting glucose metabolism representing a plausible underlying biological pathway. Although causal relationships and differential toxicity across particle fractions cannot be definitively established due to the study’s observational nature, our findings contribute valuable epidemiological evidence linking ambient air pollution to altered fetal growth dynamics. These results may support public health strategies aimed at mitigating maternal PM exposure to safeguard early-life development.

## Figures and Tables

**Table 1:** The general characteristics of the study population (*n* = 616).

	Total Population
Maternal characteristics during pregnancy (*n* = 616)	
Maternal age at enrollment, mean ± SD	30.54 ± 4.87
Maternal employment ^a^, *n* (%)	
No	167 (28.55)
Yes	418 (71.45)
Education level, *n* (%)	
Senior high school or below	226 (36.69)
Junior college	178 (28.90)
University or above	212 (34.42)
Monthly household income, *n* (%)	
<4000 RMB	267 (43.34)
4000–8000 RMB	209 (33.93)
≥8000 RMB	140 (22.73)
Pre-pregnancy BMI ^a^, *n* (%)	
<23.9 kg/m^2^	528 (88.00)
≥24 kg/m^2^	72 (12.00)
Physical activity (METs·h/w) ^a^, mean ± SD	31.45 ± 27.08
Dietary energy intake ^a^, kcal/day, mean ± SD	1825.38 ± 487.12
Maternal passive smoking ^a,b^, *n* (%)	
No	471 (79.29)
Yes	123 (20.71)
Parity, *n* (%)	
Primiparity	317 (51.46)
Multiparity	299 (48.54)
Season of conception, *n* (%)	
Summer-Autumn	509 (82.63)
Winter-Spring	107 (17.37)
Fetal sex, *n* (%)	
Male	299 (48.54)
Female	317 (51.46)
Environmental exposure during pregnancy (*n* = 616)	
PM_1_, μg/m^3^, mean ± SD	17.67 ± 2.40
PM_2.5_, μg/m^3^, mean ± SD	34.47 ± 3.67
PM_10_, μg/m^3^, mean ± SD	57.25 ± 5.21
Average temperature, °C, mean ± SD	23.38 ± 0.23
NO_2_, μg/m^3^, mean ± SD	57.48 ± 6.66
O_3_, μg/m^3^, mean ± SD	89.06 ± 8.85
Maternal glucose at mid-pregnancy (*n* = 595/510) ^c^	
FPG, mmol/L, mean ± SD	4.40 ± 0.40
OGTT 1 h glucose, mmol/L, mean ± SD	7.88 ± 1.73
OGTT 2 h glucose, mmol/L, mean ± SD	6.79 ± 1.32
Insulin, μU/mL, mean ± SD	4.36 ± 1.98
HOMA-IR, mean ± SD	0.86 ± 0.46
Birth outcomes (*n* = 573)	
Birth weight, g, mean ± SD	3192.42 ± 372.87
LGA, *n* (%)	23 (4.01)
SGA, *n* (%)	93 (16.23)

Abbreviations: SD, standard deviation; BMI, body mass index; METs, metabolic equivalents; PM_1_, particulate matter with aerodynamic diameters ≤ 1 μm; PM_2.5_, particulate matter with aerodynamic diameters ≤ 2.5 μm; PM_10_, particulate matter with aerodynamic diameters ≤ 10 μm; NO_2_, nitrogen dioxide; O_3_, ozone; FPG, fasting plasma glucose; OGTT, oral glucose tolerance test; HOMA-IR, homeostatic model assessment for insulin resistance; LGA, large for gestational age; SGA, small for gestational age. ^a^ Missing data existed for certain maternal characteristics, including 2.6% (*n* = 16) for pre-pregnancy BMI, 3.2% (*n* = 20) for physical activity, 3.6% (*n* = 22) for passive smoking, 5.0% (*n* = 31) for maternal employment, and 7.1% (*n* = 44) for dietary energy intake. ^b^ Maternal passive smoking was defined as being exposed to secondhand smoke for more than 15 min per day for at least 1 day per week during pregnancy (yes or no). ^c^ A total of 595 underwent OGTT, and 510 measured insulin and HOMA-IR. Data are presented as mean ± SD or *n* (%).

**Table 2:** Mediation analyses of maternal glucose biomarkers in the association between PM exposure and rapid EFW growth trajectory during pregnancy.

	Total Effect ^a^ [OR (95%CI)]	Direct Effect ^a^ [OR (95%CI)]	Indirect Effect ^a^ [OR (95%CI)]	Proportion ^b^ Mediated
FPG				
PM_1_	1.047 (1.004, 1.095)	1.043 (1.002, 1.092)	1.004 (0.998, 1.011)	-
PM_2.5_	1.055 (1.014, 1.103)	1.049 (1.010, 1.097)	1.006 (1.000, 1.013)	10.3%
PM_10_	1.054 (1.011, 1.104)	1.047 (1.005, 1.097)	1.007 (1.001, 1.015)	12.5%
Insulin				
PM_1_	1.293 (1.017, 1.649)	1.239 (0.976, 1.586)	1.044 (1.001, 1.101)	16.0%
PM_2.5_	1.239 (1.040, 1.487)	1.209 (1.020, 1.451)	1.025 (0.993, 1.063)	-
PM_10_	1.048 (1.003, 1.100)	1.037 (0.993, 1.088)	1.010 (1.002, 1.022)	21.9%
HOMA-IR				
PM_1_	1.048 (1.003, 1.102)	1.040 (0.995, 1.094)	1.007 (1.000, 1.020)	15.7%
PM_2.5_	1.053 (1.006, 1.103)	1.047 (1.001, 1.096)	1.006 (0.999, 1.017)	-
PM_10_	1.048 (1.001, 1.101)	1.037 (0.992, 1.089)	1.010 (1.002, 1.024)	22.2%

Abbreviations: PM_1_, particulate matter with aerodynamic diameters ≤ 1 μm; PM_2.5_, particulate matter with aerodynamic diameters ≤ 2.5 μm; PM_10_, particulate matter with aerodynamic diameters ≤ 10 μm; EFW, estimated fetal weight; OR, odds ratio; FPG, fasting plasma glucose; HOMA-IR, homeostatic model assessment for insulin resistance. ^a^ Estimates for the rapid growth trajectory (average growth trajectory as reference) are reported per standard-deviation increase in PM exposure in the logistic regression-based mediation analysis. ^b^ Mediation proportions were shown if indirect effects are significant. The proportion mediated was calculated within each bootstrap sample (2000 resamples) as the indirect effect divided by the total effect, and the mean of the bootstrap-specific proportions was reported. The model was adjusted for maternal age, maternal employment status, household income, maternal education, physical activity, pre-pregnancy BMI, parity, fetal sex, season of conception, and average temperature during pregnancy. Maternal employment status, pre-pregnancy BMI, and physical activity had missing data for 5.0% (*n* = 31), 2.6% (*n* = 16), and 3.2% (*n* = 20), which were handled with the Multivariate Imputation by Chained Equations algorithm.

**Table 3:** Mediation analyses of maternal glucose biomarkers in associations of PM exposure with fetal growth z-scores.

Outcomes/ Mediator	Exposure	Total Effect [β (95%CI)]	Direct Effect [β (95%CI)]	Indirect Effect [β (95%CI)]	Proportion Mediated ^a^
EFW z-score					
FPG	PM_1_	0.116 (0.048, 0.179)	0.103 (0.035, 0.166)	0.013 (0.005, 0.023)	12.7%
	PM_2.5_	0.064 (0.026, 0.102)	0.056 (0.017, 0.093)	0.008 (0.003, 0.015)	14.8%
	PM_10_	0.047 (0.017, 0.076)	0.040 (0.01, 0.069)	0.007 (0.003, 0.012)	15.3%
Insulin	PM_1_	0.108 (0.039, 0.181)	0.091 (0.020, 0.162)	0.017 (0.007, 0.032)	17.6%
	PM_2.5_	0.058 (0.017, 0.098)	0.051 (0.010, 0.090)	0.007 (0.002, 0.013)	12.5%
	PM_10_	0.040 (0.008, 0.071)	0.031 (0.000, 0.062)	0.008 (0.004, 0.014)	29.2%
HOMA-IR	PM_1_	0.107 (0.037, 0.180)	0.091 (0.02, 0.162)	0.016 (0.006, 0.031)	17.3%
	PM_2.5_	0.057 (0.015, 0.097)	0.050 (0.008, 0.092)	0.007 (0.002, 0.013)	14.4%
	PM_10_	0.038 (0.006, 0.070)	0.031 (−0.001, 0.062)	0.008 (0.004, 0.014)	28.8%
BPD z-score					
FPG	PM_1_	0.085 (0.015, 0.150)	0.077 (0.007, 0.142)	0.008 (0.002, 0.017)	11.7%
	PM_2.5_	0.055 (0.016, 0.092)	0.049 (0.010, 0.086)	0.005 (0.001, 0.011)	13.8%
	PM_10_	0.039 (0.008, 0.067)	0.034 (0.004, 0.063)	0.004 (0.001, 0.009)	14.3%
Insulin	PM_1_	0.079 (0.005, 0.150)	0.072 (−0.004, 0.142)	0.007 (0.000, 0.016)	7.4%
	PM_2.5_	0.050 (0.008, 0.091)	0.047 (0.006, 0.088)	0.003 (0.000, 0.007)	7.5%
	PM_10_	0.031 (0.000, 0.062)	0.028 (−0.003, 0.059)	0.003 (0.000, 0.007)	24.9%
HOMA-IR	PM_1_	0.080 (0.007, 0.155)	0.072 (−0.002, 0.148)	0.008 (0.001, 0.016)	16.5%
	PM_2.5_	0.050 (0.009, 0.092)	0.047 (0.006, 0.088)	0.003 (0.000, 0.007)	12.8%
	PM_10_	0.031 (0.000, 0.063)	0.027 (−0.003, 0.059)	0.004 (0.001, 0.007)	18.7%
HC z-score					
FPG	PM_1_	0.107 (0.038, 0.173)	0.100 (0.031, 0.165)	0.008 (0.001, 0.016)	8.2%
	PM_2.5_	0.062 (0.024, 0.099)	0.057 (0.018, 0.094)	0.005 (0.001, 0.010)	8.9%
	PM_10_	0.044 (0.015, 0.072)	0.039 (0.009, 0.068)	0.004 (0.001, 0.008)	9.1%
Insulin	PM_1_	0.089 (0.019, 0.160)	0.079 (0.008, 0.15)	0.009 (0.002, 0.019)	11.9%
	PM_2.5_	0.050 (0.010, 0.091)	0.047 (0.006, 0.087)	0.004 (0.001, 0.008)	9.2%
	PM_10_	0.030 (0.000, 0.061)	0.026 (−0.004, 0.057)	0.004 (0.001, 0.009)	14.0%
HOMA-IR	PM_1_	0.088 (0.012, 0.16)	0.079 (0.003, 0.153)	0.009 (0.001, 0.019)	10.8%
	PM_2.5_	0.050 (0.007, 0.092)	0.046 (0.004, 0.089)	0.004 (0.001, 0.008)	5.0%
	PM_10_	0.03 (−0.001, 0.061)	0.026 (−0.005, 0.058)	0.004 (0.001, 0.009)	15.4%
AC z-score					
FPG	PM_1_	0.113 (0.050, 0.178)	0.102 (0.038, 0.165)	0.012 (0.004, 0.021)	11.5%
	PM_2.5_	0.061 (0.025, 0.097)	0.053 (0.016, 0.090)	0.008 (0.003, 0.014)	15.4%
	PM_10_	0.043 (0.016, 0.071)	0.037 (0.009, 0.065)	0.006 (0.002, 0.011)	13.9%
Insulin	PM_1_	0.118 (0.045, 0.191)	0.099 (0.027, 0.173)	0.018 (0.007, 0.032)	17.6%
	PM_2.5_	0.062 (0.023, 0.102)	0.055 (0.016, 0.096)	0.007 (0.002, 0.014)	12.6%
	PM_10_	0.043 (0.011, 0.074)	0.034 (0.003, 0.066)	0.008 (0.004, 0.014)	29.4%
HOMA-IR	PM_1_	0.117 (0.048, 0.190)	0.100 (0.032, 0.172)	0.017 (0.006, 0.033)	15.9%
	PM_2.5_	0.062 (0.020, 0.104)	0.055 (0.012, 0.096)	0.007 (0.002, 0.014)	13.5%
	PM_10_	0.042 (0.011, 0.074)	0.034 (0.002, 0.065)	0.008 (0.004, 0.014)	25.2%

Abbreviations: PM_1_, particulate matter with aerodynamic diameters ≤ 1 μm; PM_2.5_, particulate matter with aerodynamic diameters ≤ 2.5 μm; PM_10_, particulate matter with aerodynamic diameters ≤ 10 μm; FPG, fasting plasma glucose; HOMA-IR, homeostatic model assessment for insulin resistance; BPD: biparietal diameter; HC, head circumference; AC, abdominal circumference; EFW, estimated fetal weight. Effect estimates are reported per 5-μg/m^3^ increase in exposure. ^a^ Mediation proportions were shown if indirect effects are significant. The proportion mediated was calculated within each bootstrap sample (2000 resamples) as the indirect effect divided by the total effect, and the mean of the bootstrap-specific proportions was reported. The generalized estimating equations (GEE)-based mediation model was adjusted for gestational age, maternal age, maternal employment status, household income, maternal education, physical activity, pre-pregnancy BMI, parity, fetal sex, season of conception, and average temperature during pregnancy. Maternal employment status, pre-pregnancy BMI, and physical activity had missing data for 5.0% (*n* = 31), 2.6% (*n* = 16), and 3.2% (*n* = 20), which were handled with the Multivariate Imputation by Chained Equations algorithm.

## Data Availability

The raw data supporting the conclusions of this article will be made available by the authors on request.

## Supplementary Materials

The following supporting information can be downloaded at: https://www.mdpi.com/article/10.3390/toxics14080709/s1, Figure S1: The spatial distribution of pregnant women recruited from 2017 to 2018 in Guangzhou; Figure S2: The estimation of latent class mixed models (LCMM) with different numbers of latent classes for fetal estimated weight (EFW) trajectories; Figure S3: Directed acyclic graph for the hypothesized relationship between maternal PM exposure and fetal growth; Figure S4: The pairwise Spearman correlation coefficients of PM and other environmental exposures during pregnancy (*n* = 616); Figure S5: The dose–response relationship between PM exposure during pregnancy and rapid growth trajectory of EFW (reference: average growth class, *n* = 569); Figure S6: The relationship between PM exposure during pregnancy and EFW z-score at each measurement (*n* = 585/601/592); Figure S7: The associations between (A) fetal growth trajectories in utero and (B) prenatal PM exposure (per 5 μg/m^3^) with LGA and SGA at birth; Table S1: Posterior probabilities of 3-latent class model for fetal estimated weight (EFW) trajectories and association with birth weight z-score; Table S2: Description of covariates after imputation; Table S3: Description of maternal PM exposure and repeated measurements of fetal growth parameters during pregnancy; Table S4: The longitudinal association between maternal exposure to PM (per 5 μg/m^3^) and fetal growth parameters in utero in the GEE model (*n* = 616); Table S5: The association of maternal glucose hemostasis biomarkers at mid-pregnancy and the trajectories of EFW in utero; Table S6: The association of maternal glucose hemostasis biomarkers at mid-pregnancy and fetal growth parameters in utero in the GEE model; Table S7: The association between maternal exposure to PM (per 5 μg/m^3^) and fetal growth in utero with adjustment for additional risk factors for outcomes; Table S8: The pooled association between maternal exposure to PM (per 5 μg/m^3^) and fetal growth in utero with multiple imputed datasets (imputed number = 5); Table S9: The longitudinal association between maternal exposure to size-specific PM (per 5 μg/m^3^) and overgrowth risk (z-score > 1.88) in utero in the GEE model; Table S10: The associations between fetal growth trajectories in utero and prenatal PM exposure (per 5 μg/m^3^) with birth weight z-score; Table S11: Mediation analyses of maternal glucose biomarkers in the association between maternal PM exposure and fetal growth outcomes in the additionally adjusted model; Table S12: Mediation analyses of maternal glucose biomarkers in the longitudinal association between PM (per 5 μg/m^3^) and z-scores of fetal parameters in utero under a stricter temporal sequence, with PM exposure restricted to the period before glucose assessment and fetal growth measurements restricted to those obtained thereafter.

## Abbreviations

PM	Particulate Matter
HC	Head Circumference
AC	Abdominal circumference
FL	Femur Length
BPD	Biparietal Diameter
EFW	Estimated Fetal Weight
GDM	Gestational Diabetes Mellitus
LMP	Last Menstrual Period
LGA	Large for Gestational Age
SGA	Small for Gestational Age
CHAP	China High Air Pollutants
CV-R^2^	Cross-Validation Coefficients of Determination
RMSE	Root Mean Square Error
OGTT	Oral Glucose Tolerance Test
FPG	Fasting Plasma Glucose
HOMA-IR	Homeostasis Model Assessment of Insulin Resistance
NO_2_	Nitrogen Dioxide
O_3_	Ozone
SD	Standard Deviation
LCMM	Latent Class Mixed Model
GEE	Generalized Estimating Equation
