# Supplementary material for: Prenatal Exposure to Size-Fractionated Particulate Matter and Fetal Growth Trajectories: Mediation by Maternal Glucose Metabolism in a Birth Cohort
Source: Toxics. 2026 Aug 12;14(8):709. doi: 10.3390/toxics14080709 (PMC13517812; doi:10.3390/toxics14080709)
Supplement: Supplementary file 1 [file toxics-14-00709-s001.zip › toxics-4428689-supplementary.pdf]

Supporting Information

Contents

Figure S1. The spatial distribution of pregnant women recruited from 2017 to 2018 in Guangzhou.

Figure S2. The estimation of latent class mixed models (LCMM) with different numbers of latent classes for fetal estimated weight (EFW) trajectories.

Table S1. Posterior probabilities of 3-latent class model for fetal estimated weight (EFW) trajectories and association with birth weight z-score.

Figure S3. Directed acyclic graph for the hypothesized relationship between maternal PM exposure and fetal growth.

Table S2. Description of covariates after imputation.

Figure S4. The pairwise Spearman correlation coefficients of PM and other environmental exposures during pregnancyy(n=616).

Table S3. Description of maternal PM exposure and repeated measurements of fetal growth parameters during pregnancy.

Table S4. The longitudinal association between maternal exposure to PM (per 5 $\mu g/m^3$) and fetal growth parameters in utero in the GEE model (n=616).

Figure S5. The dose–response relationship between PM exposure during pregnancy and rapid growth trajectory of EFW (reference: average growth class, n=569).

Figure S6. The relationship between PM exposure during pregnancy and EFW z-score at each measurement (n=585 / 601 / 592).

Table S5. The association of maternal glucose hemostasis biomarkers at mid-pregnancy and the trajectories of EFW in utero.

Table S6. The association of maternal glucose hemostasis biomarkers at mid-pregnancy and fetal growth parameters in utero in the GEE model.

Table S7. The association between maternal exposure to PM (per 5 $\mu g/m^3$) and fetal growth in utero with adjustment for additional risk factors for outcomes.

Table S8. The pooled association between maternal exposure to PM (per 5 $\mu g/m^3$) and fetal growth in utero with multiple imputed datasets (imputed number=5).

Table S9. The longitudinal association between maternal exposure to size-specific PM (per 5 $\mu g/m^3$) and overgrowth risk (z-score > 1.88) in utero in the GEE model.

Table S10. The associations between fetal growth trajectories in utero and prenatal PM exposure (per 5 $\mu g/m^3$) with birth weight z-score.

Figure S7. The associations between (A) fetal growth trajectories in utero and (B) prenatal PM exposure (per 5 $\mu g/m^3$) with LGA and SGA at birth.

Table S11. Mediation analyses of maternal glucose biomarkers in the association between maternal PM exposure and fetal growth outcomes in the additionally adjusted model.

Table S12. Mediation analyses of maternal glucose biomarkers in the longitudinal association between PM (per 5 $\mu g/m^3$) and z-scores of fetal parameters in utero under a stricter temporal sequence, with PM exposure restricted to the period before glucose assessment and fetal growth measurements restricted to those obtained thereafter.

Supplementary materials

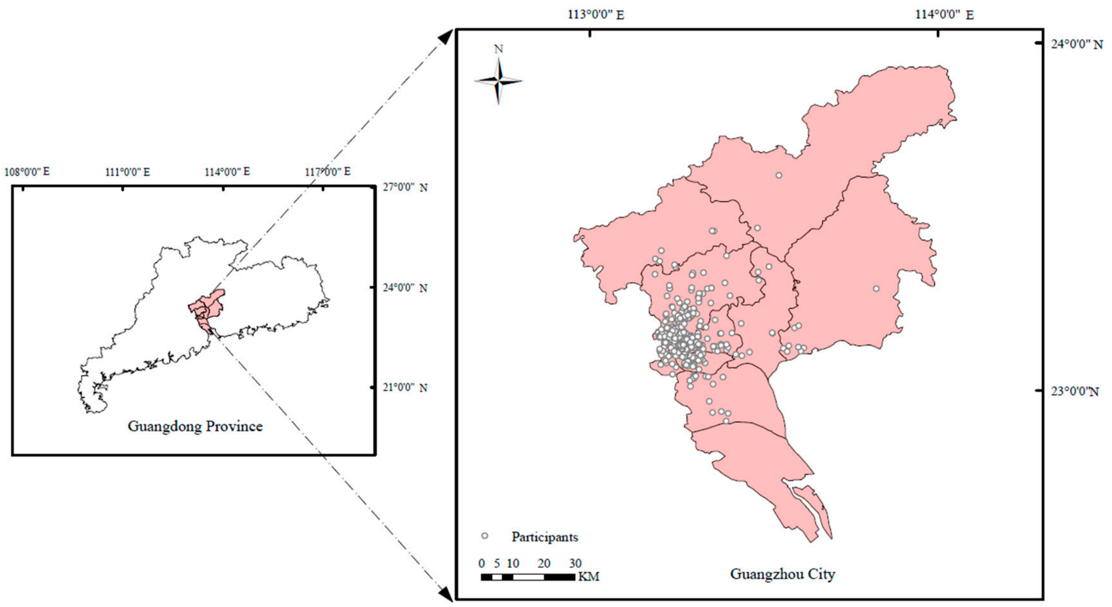

Figure S1. The spatial distribution of pregnant women recruited from 2017 to 2018 in Guangzhou, China (n=616).

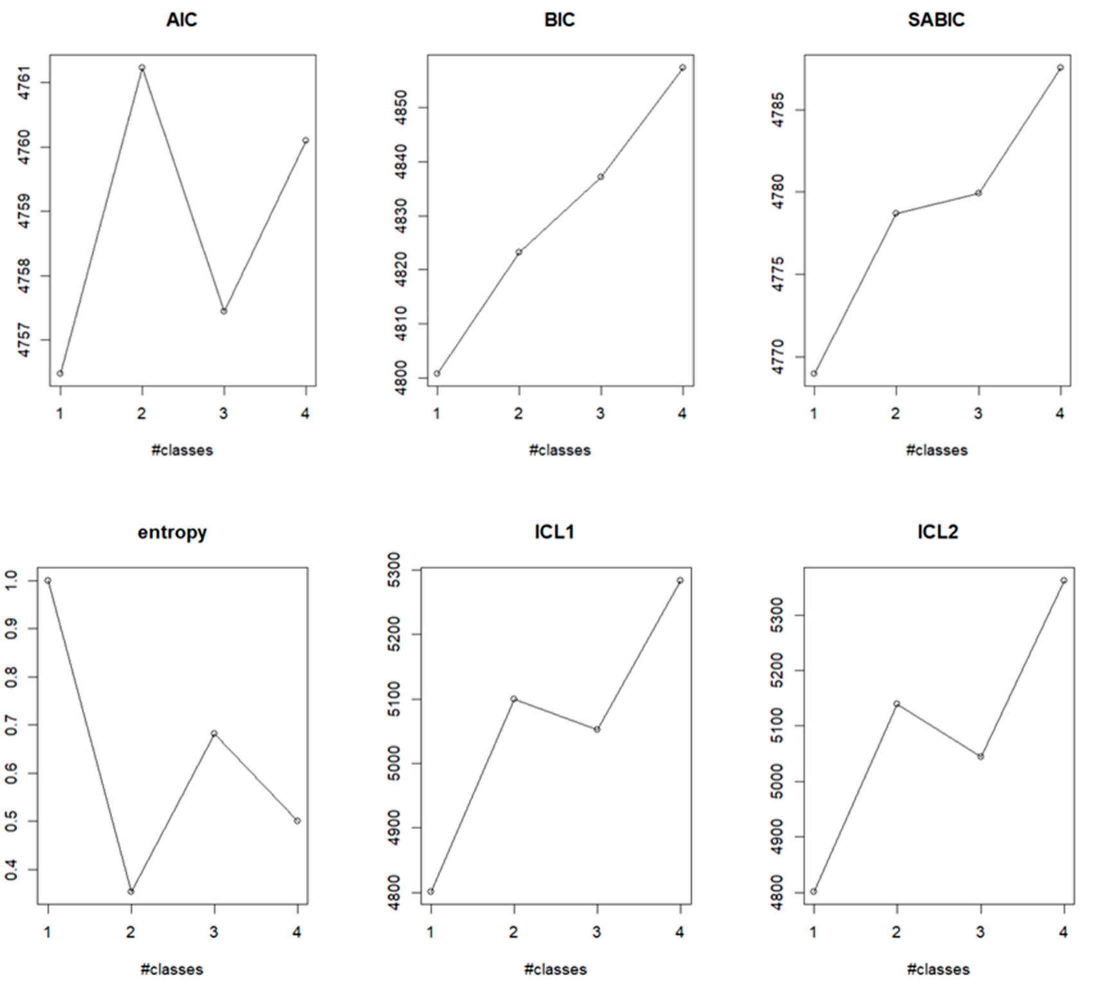

Figure S2. The estimation of latent class mixed models (LCMM) with different numbers of latent classes for fetal estimated weight (EFW) trajectories.

Abbreviations: AIC, Akaike Information Criterion; BIC, Bayesian Information Criterion; ASBIC, Adjusted Sample-Size Bayesian Information Criterion; ICL1, Integrated Complete Likelihood Criterion (Version 1); ICL2, Integrated Complete Likelihood Criterion (Version 2).

AIC, BIC, ASBIC, ICL1, and ICL2: the lower the better.

Entropy: the closer to one, the better.

Table S1. Posterior probabilities of 3-latent class model for fetal estimated weight (EFW) trajectories.

	%	Posterior probabilities in each class			Posterior probabilities
		Class 1	Class 2	Class 3	>0.70
Class 1	68.18	86.94	5.25	7.8	88.1
Class 2	7.63	20.69	79.3	0.01	61.7
Class 3	24.19	15.51	0	84.49	79.87

Class 1: average growth; Class 2: slow growth; Class 3: rapid growth.

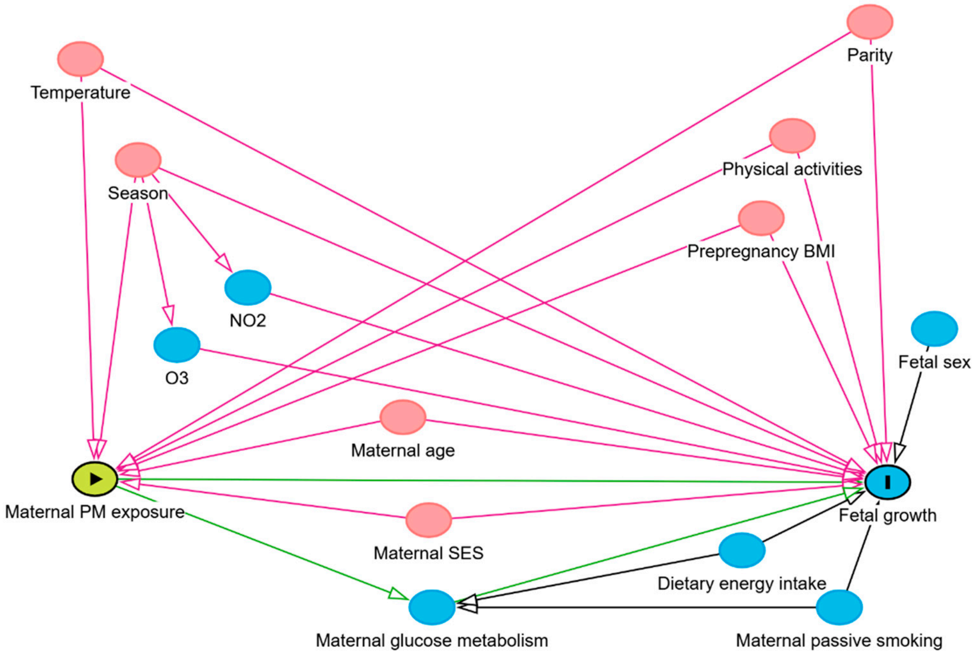

Figure S3. Directed acyclic graph for the hypothesized relationship between maternal PM exposure and fetal growth.

Abbreviations: PM, particulate matter; SES: socioeconomic status, including maternal employment, maternal education level, and household income.

Green arrows indicate the hypothesized exposure–mediator–outcome pathway, pink arrows indicate potential exposure–outcome biasing pathways, and black arrows indicate additional determinants of maternal glucose metabolism or fetal growth. The primary adjustment set was prespecified for the PM–fetal growth association, while dietary energy intake, passive smoking, NO_2, and O_3 were further evaluated in expanded sensitivity models. The graph was created using DAGitty version 3.1.

Table S2. Description of covariates after imputation (n=616).

	Original dataset	Imputed covariates (imputed datasets=5)					
		1	2	3	4	5	p
Maternal employment, yes, n (%)	418 (71.45)	440 (71.43)	441 (71.59)	438 (71.10)	449 (72.89)	440 (71.43)	0.988
N-Miss	31	0	0	0	0	0	
Pre-pregnancy BMI, n (%)							1.000
<23.9 kg/m^2	528 (88.00)	541 (87.82)	543 (88.15)	542 (87.99)	543 (88.15)	542 (87.99)	
≥24 kg/m^2	72 (12.00)	75 (12.18)	73 (11.85)	74 (12.01)	73 (11.85)	74 (12.01)	
N-Miss	16	0	0	0	0	0	
Physical activity (METs ·h/w)							0.993
Mean ± SD	31.45 ± 27.08	31.76 ± 27.33	31.39 ± 26.81	31.53 ± 26.76	32.14 ± 27.18	31.18 ± 26.73	
N-Miss	20	0	0	0	0	0	
Maternal passive smoking, yes, n	123 (20.71)	128 (20.78)	133 (21.59)	123 (19.97)	123 (19.97)	128 (20.78)	0.983
N-Miss	22	0	0	0	0	0	
Dietary energy intake, kcal							0.972
Mean ± SD	1825.28 ± 486.70	1806.41 ± 486.65	1827.75 ± 492.24	1816.78 ± 482.49	1819.17 ± 484.60	1810.51± 485.39	
N-Miss	44	0	0	0	0	0	

Data were presented as mean ± SD or n (%).

P values from chi-square test or ANOVA test.

Missing data were imputed with Multivariate Imputation by Chained Equations algorithm (imputed number=5 and iteration=10).

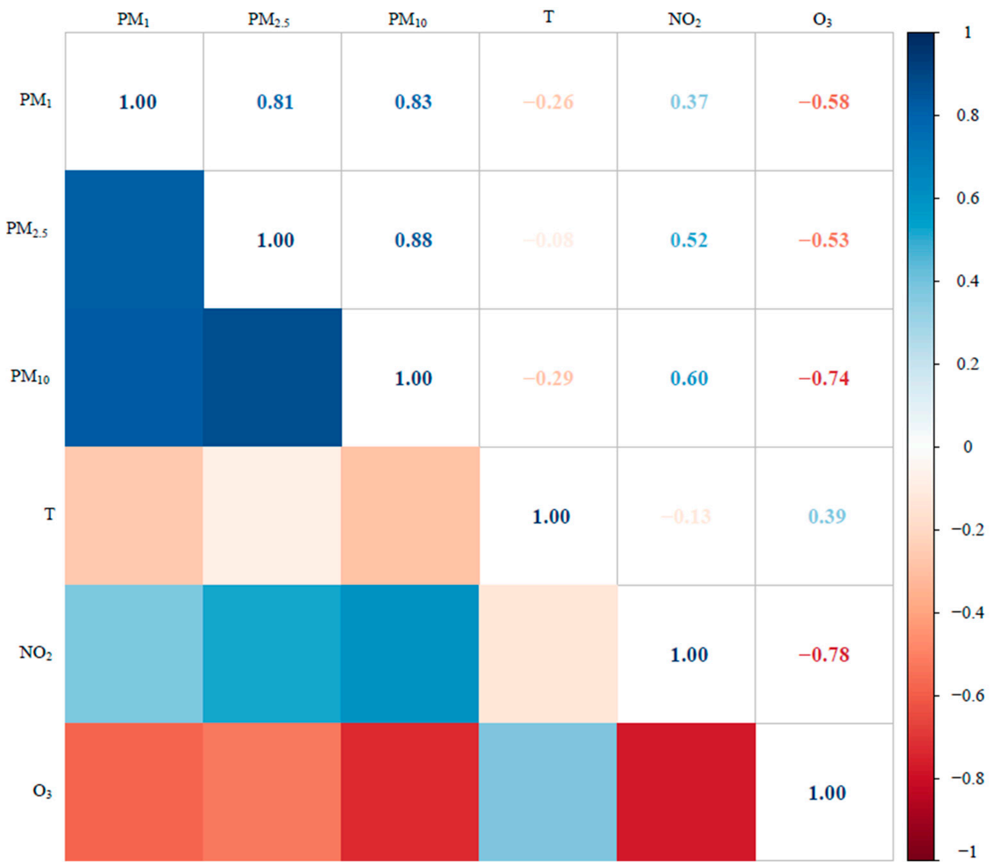

Figure S4. The pairwise Spearman correlation coefficients of PM and other environmental exposures during pregnancy (n=616).

Abbreviations: PM_1, particulate matter with aerodynamic diameters ≤ 1μm, $PM_{2.5}$; particulate matter with aerodynamic diameters ≤ 2.5μm; PM_{10}, particulate matter with aerodynamic diameters ≤ 10 μm; T, temperature; NO_2, nitrogen dioxide; O_3, ozone.

Table S3. Description of maternal PM exposure and repeated measurements of fetal growth parameters during pregnancy.

	Measurement 1 (n=585)	Measurement 2 (n=601)	Measurement 3 (n=592)
Gestational weeks	22.30 ±1.83	29.44 ± 1.36	36.11 ± 1.09
PM exposure, mean ±SD			
PM_1 (μg/m^3)	20.17 ± 4.23	18.85 ± 3.68	17.95 ± 2.87
$PM_{2.5}$ (μg/m^3)	39.98 ± 7.43	36.55 ± 6.27	34.96 ± 4.60
PM_{10} (μg/m^3)	63.28 ± 9.88	60.15 ± 8.63	57.93 ± 6.44
Fetal growth			
Original scale, mean ±SD			
BPD (mm)	53.94 ± 6.20	75.09 ± 4.31	89.07 ± 3.22
HC (mm)	200.71 ± 21.90	274.48 ± 13.58	319.63 ± 9.61
AC (mm)	176.36 ± 20.84	253.72 ± 17.57	320.17 ± 14.00
FL (mm)	38.60 ± 5.26	56.04 ± 3.45	68.90 ± 2.55
EFW (g)	522.99 ± 157.23	1441.13 ± 280.55	2764.93 ± 299.94
Z-score, mean ±SD			
BPD	-0.01 ± 0.98	0.02 ± 1.01	-0.01 ± 1.01
HC	-0.02 ± 0.99	0.03 ± 1.00	0.00 ± 0.99
AC	-0.04 ± 0.96	0.02 ± 1.01	0.03 ± 0.99
FL	-0.04 ± 0.99	0.03 ± 1.02	0.01 ± 0.99
EFW	-0.04 ± 0.97	0.02 ± 1.02	0.02 ± 0.98
Fetal overgrowth, n (%)			
BPD	13 (2.22)	20 (3.33)	23 (3.89)
HC	15 (2.56)	18 (3.00)	17 (2.88)
AC	11 (1.88)	18 (3.00)	24 (4.05)
FL	16 (2.74)	20 (3.33)	19 (3.21)
EFW	11 (1.88)	17 (2.83)	17 (2.88)

Abbreviations: PM_1, particulate matter with aerodynamic diameters ≤ 1μm; $PM_{2.5}$, particulate matter with aerodynamic diameters ≤ 2.5μm; PM_{10}, particulate matter with aerodynamic diameters ≤ 10 μm.; BPD: biparietal diameter; HC, head circumference; AC, abdominal circumference; FL, femur length; EFW, estimated fetal weight.

A total of 616 women had at least one measurement during pregnancy. The number of participants measured three times, twice, and once are 556 (90.26%), 50 (8.12%), and 10 (1.62%).

Overgrowth: z-score>1.88 (97th centile).

Table S4. The longitudinal association between maternal exposure to PM (per 5 μg/m^3) and fetal growth parameters in utero in the GEE model (n=616).

	Crude model			Main model		
	β (95%CI)	*p*	FDR	β (95%CI)	*p*	FDR
PM_1						
BPD	0.064 (0.001,0.127)	0.045	0.056	0.075 (0.008, 0.143)	0.029	0.036
HC	0.097 (0.035,0.160)	0.002	0.005	0.102 (0.036, 0.168)	0.003	0.007
AC	0.107 (0.046,0.168)	0.001	0.004	0.105 (0.040, 0.169)	0.002	0.007
FL	0.051 (-0.013,0.115)	0.116	0.121	0.056 (-0.012, 0.125)	0.106	0.106
EFW	0.108 (0.047,0.170)	0.001	0.004	0.107 (0.042, 0.173)	0.001	0.007
$PM_{2.5}$						
BPD	0.049 (0.012,0.085)	0.010	0.014	0.050 (0.012, 0.088)	0.010	0.015
HC	0.057 (0.020,0.094)	0.003	0.005	0.059 (0.020, 0.097)	0.003	0.007
AC	0.056 (0.021,0.091)	0.002	0.005	0.057 (0.021, 0.094)	0.002	0.007
FL	0.032 (-0.006,0.069)	0.097	0.112	0.035 (-0.004, 0.074)	0.076	0.088
EFW	0.059 (0.023,0.095)	0.001	0.005	0.060 (0.023, 0.098)	0.002	0.007
PM_{10}						
BPD	0.029 (0.002,0.056)	0.034	0.047	0.035 (0.006, 0.064)	0.017	0.023
HC	0.038 (0.011,0.065)	0.006	0.011	0.040 (0.011, 0.069)	0.006	0.010
AC	0.041 (0.015,0.067)	0.002	0.005	0.040 (0.012, 0.068)	0.005	0.010
FL	0.022 (-0.006,0.049)	0.121	0.121	0.025 (-0.005, 0.054)	0.098	0.105
EFW	0.043 (0.016,0.069)	0.002	0.005	0.043 (0.014, 0.071)	0.003	0.007

Abbreviations: GEE, generalized estimating equations; PM_1, particulate matter with aerodynamic diameters ≤ 1μm; $PM_{2.5}$, particulate matter with aerodynamic diameters ≤ 2.5μm; PM_{10}, particulate matter with aerodynamic diameters ≤ 10 μm; BPD: biparietal diameter; HC, head circumference; AC, abdominal circumference; FL, femur length; EFW, estimated fetal weight. FDR, false discovery rate.

The crude model was adjusted for nothing.

The main GEE model was adjusted for gestational age, maternal age, maternal employment status, household income, maternal education, physical activity, pre-pregnancy BMI, parity, fetal sex, season of conception, and average temperature during pregnancy.

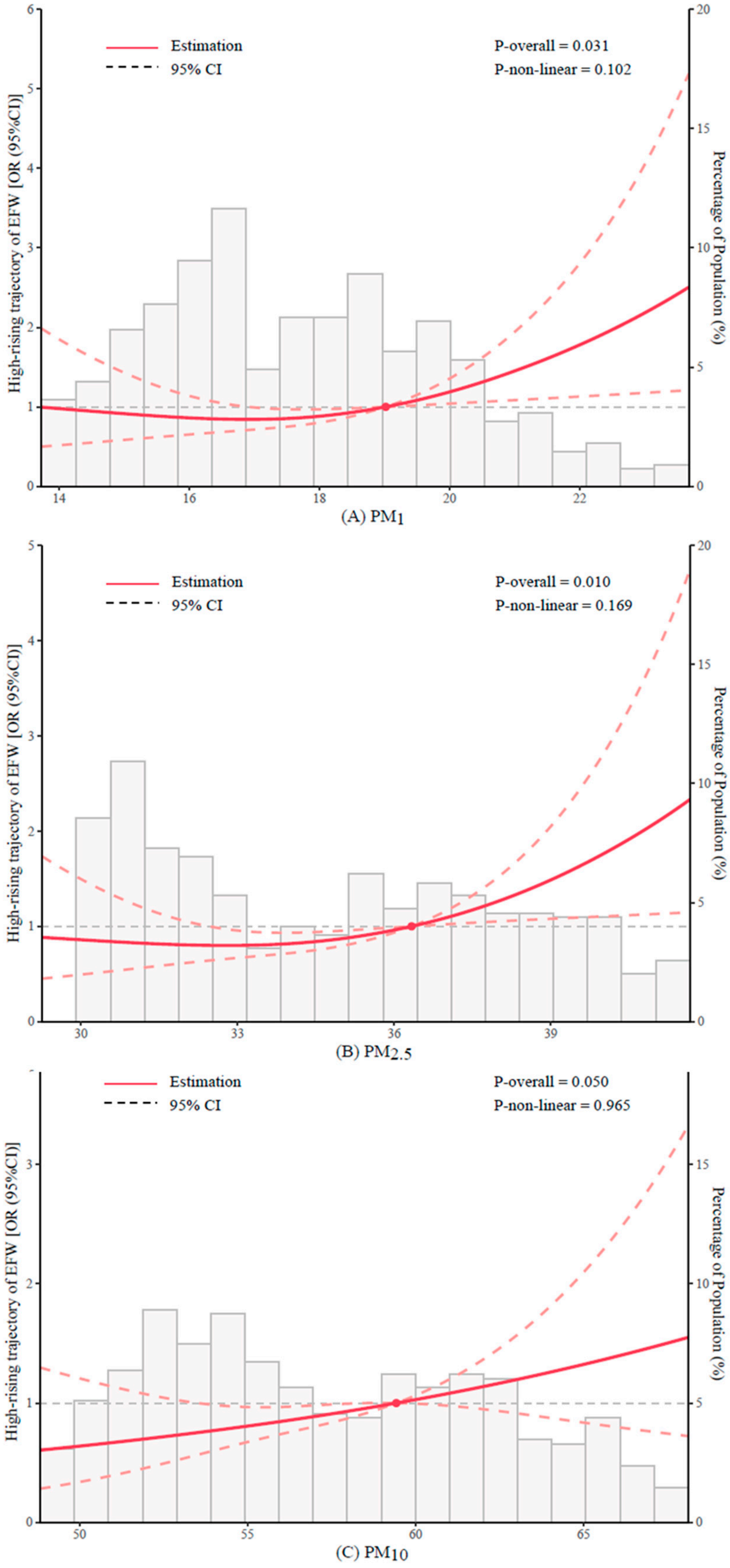

Figure S5. The dose–response relationship between PM exposure during pregnancy and rapid growth trajectory of EFW (reference: average growth class, n=569).

Abbreviations: PM_1, particulate matter with aerodynamic diameters $\leq$ 1μm; $PM_{2.5}$, particulate matter

with aerodynamic diameters ≤ 2.5μm; PM_{10}, particulate matter with aerodynamic diameters ≤ 10 μm; EFW, estimated fetal weight; OR, odds ratio.

The model was adjusted for maternal age, maternal employment status, household income, maternal education, physical activity, pre-pregnancy BMI, parity, fetal sex, season of conception, and average temperature during pregnancy.

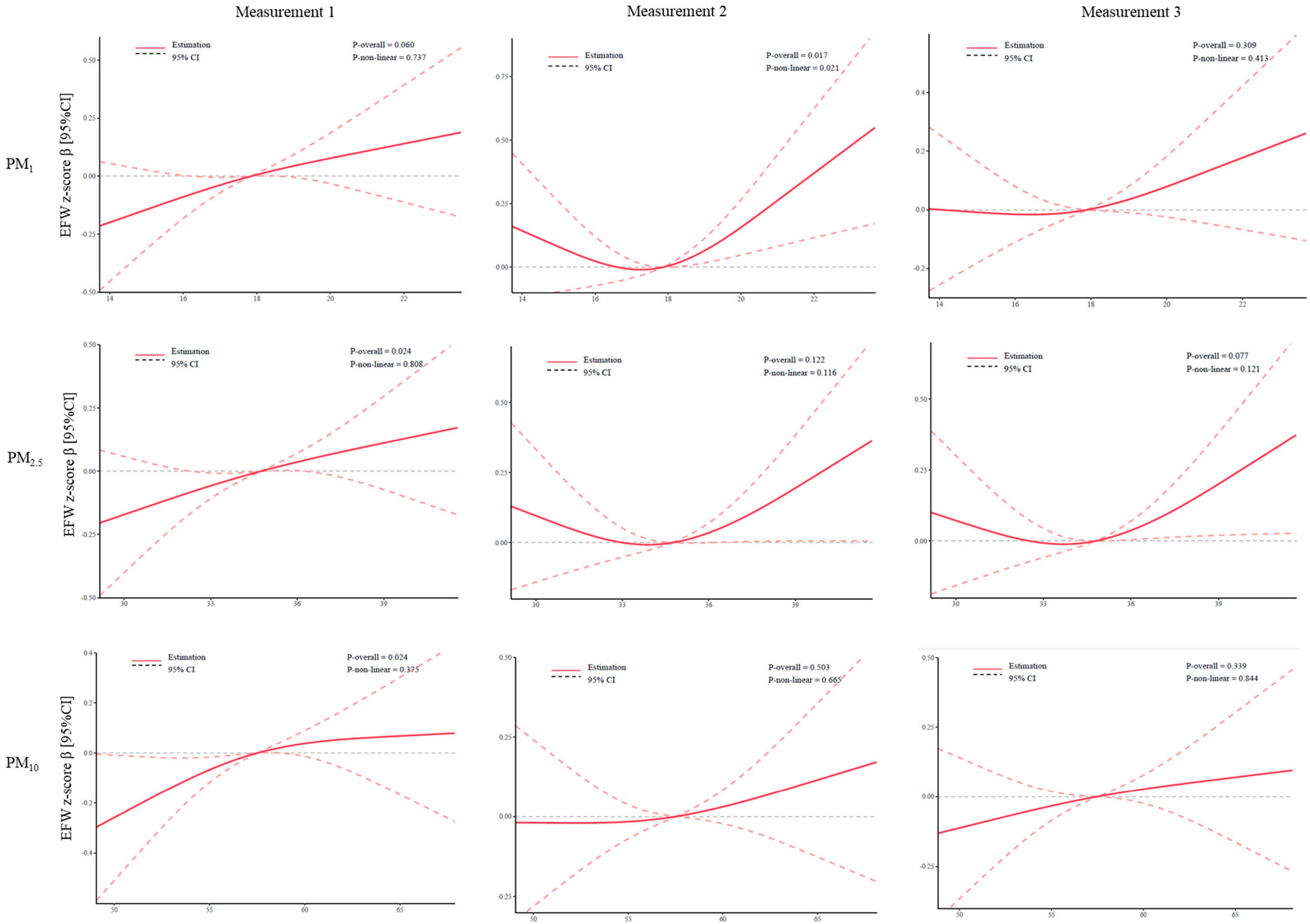

Measurement 1
Measurement 2
Measurement 3
PM1
PM2.5
PM10
EFW z-score β [95%CI]
Estimation
95% CI
P-overall = 0.060
P-non-linear = 0.737
P-overall = 0.017
P-non-linear = 0.021
P-overall = 0.309
P-non-linear = 0.413
P-overall = 0.024
P-non-linear = 0.808
P-overall = 0.122
P-non-linear = 0.116
P-overall = 0.077
P-non-linear = 0.121
P-overall = 0.024
P-non-linear = 0.375
P-overall = 0.503
P-non-linear = 0.665
P-overall = 0.339
P-non-linear = 0.844

Figure S6. The relationship between PM exposure during pregnancy and EFW z-score at each measurement (n=585 / 601 / 592).

Abbreviations: PM_1, particulate matter with aerodynamic diameters ≤ 1μm; $PM_{2.5}$, particulate matter with aerodynamic diameters ≤ 2.5μm; PM_{10}, particulate matter with aerodynamic diameters ≤ 10 μm; EFW, estimated fetal weight; OR, odds ratio.

The model was adjusted for gestational age, maternal age, maternal employment status, household income, maternal education, physical activity, pre-pregnancy BMI, parity, fetal sex, season of conception, and average temperature during pregnancy.

Table S5. The association of maternal glucose hemostasis biomarkers at mid-pregnancy and the trajectories of EFW in utero.

Growth trajectories [a]	OR (95%CI)	*p*
FPG		
Slow growth	0.727 (0.480, 1.101)	0.132
Rapid growth	1.567 (1.223, 2.008)	<0.001
OGTT 1 h glucose		
Slow growth	0.742 (0.479, 1.150)	0.182
Rapid growth	1.020 (0.772, 1.347)	0.889
OGTT 2 h glucose		
Slow growth	0.834 (0.548, 1.269)	0.397
Rapid growth	1.132 (0.882, 1.452)	0.331
Insulin		
Slow growth	0.632 (0.372, 1.076)	0.091
Rapid growth	1.574 (1.258, 1.971)	<0.001
HOMA-IR		
Slow growth	0.593 (0.338, 1.039)	0.068
Rapid growth	1.553 (1.236, 1.950)	<0.001

[a] The average class is the reference group. The multinomial logistic model was adjusted for maternal age, maternal employment status, household income, maternal education, physical activity, pre-pregnancy BMI, parity, gestational age, fetal sex, season of conception, and average temperature during pregnancy.

Table S6. The association of maternal glucose hemostasis biomarkers at mid-pregnancy and fetal growth parameters in utero in the GEE model.

Z-score of fetal parameters	β (95%CI)	*p*
FPG		
BPD	0.089 (0.028, 0.151)	0.004
HC	0.085 (0.024, 0.145)	0.006
AC	0.122 (0.062, 0.182)	<0.001
FL	0.118 (0.056, 0.180)	<0.001
EFW	0.135 (0.074, 0.196)	<0.001
OGTT 1 h glucose		
BPD	0.034 (-0.032, 0.099)	0.313
HC	0.023 (-0.041, 0.087)	0.483
AC	0.038 (-0.025, 0.102)	0.237
FL	0.012 (-0.054, 0.078)	0.720
EFW	0.030 (-0.033, 0.093)	0.353
OGTT 2 h glucose		
BPD	0.024 (-0.035, 0.082)	0.424
HC	0.033 (-0.026, 0.092)	0.277
AC	0.048 (-0.012, 0.108)	0.116
FL	0.002 (-0.060, 0.063)	0.961
EFW	0.036 (-0.025, 0.097)	0.247
Insulin		
BPD	0.124 (0.051, 0.197)	0.001
HC	0.061 (0.011, 0.111)	<0.001
AC	0.078 (0.023, 0.133)	<0.001
FL	0.144 (0.076, 0.211)	<0.001
EFW	0.082 (0.032, 0.133)	0.001
HOMA-IR		
BPD	0.061 (0.015, 0.106)	0.009
HC	0.068 (0.011, 0.126)	0.020
AC	0.127 (0.048, 0.206)	0.002
FL	0.080 (0.028, 0.133)	0.003
EFW	0.124 (0.051, 0.197)	0.001

The generalized linear model was adjusted for maternal age, maternal employment status, household income, maternal education, physical activity, pre-pregnancy BMI, parity, gestational age, fetal sex, season of conception, and average temperature during pregnancy.

Table S7. The association between maternal exposure to PM (**per 5 μg/m³**) and fetal growth in utero, with adjustment for additional risk factors for outcomes.

	Additional model		
	OR/β (95%CI)	*p*	FDR
**EFW trajectories** [a]			
PM_1			
Slow growth	0.360 (0.130, 1.014)	0.053	0.062
Rapid growth	1.680 (1.027, 2.760)	0.039	0.051
$PM_{2.5}$			
Slow growth	0.690 (0.389, 1.236)	0.215	0.215
Rapid growth	1.570 (1.145, 2.159)	0.005	0.008
PM_{10}			
Slow growth	0.660 (0.397, 1.110)	0.118	0.124
Rapid growth	1.470 (1.125, 1.932)	0.005	0.008
**Z-score** [b]			
PM_1			
BPD	0.108 (0.031, 0.185)	0.006	0.009
HC	0.133 (0.055, 0.211)	0.001	0.002
AC	0.130 (0.054, 0.206)	0.001	0.002
FL	0.080 (-0.002, 0.162)	0.056	0.062
EFW	0.137 (0.059, 0.214)	0.001	0.002
$PM_{2.5}$			
BPD	0.069 (0.025, 0.112)	0.002	0.004
HC	0.077 (0.032, 0.122)	0.001	0.002
AC	0.072 (0.029, 0.115)	0.001	0.003
FL	0.048 (0.001, 0.095)	0.044	0.054
EFW	0.077 (0.033, 0.122)	0.001	0.002
PM_{10}			
BPD	0.062 (0.024, 0.099)	0.001	0.003
HC	0.066 (0.028, 0.105)	0.001	0.002
AC	0.060 (0.023, 0.097)	0.001	0.003
FL	0.043 (0.004, 0.083)	0.033	0.046
EFW	0.067 (0.029, 0.105)	0.001	0.002

Abbreviations: PM_1, particulate matter with aerodynamic diameters ≤ 1μm; $PM_{2.5}$, particulate matter with aerodynamic diameters ≤ 2.5μm; PM_{10}, particulate matter with aerodynamic diameters ≤ 10 μm; BPD: biparietal diameter; HC, head circumference; AC, abdominal circumference; FL, femur length; EFW, estimated fetal weight.

[a] Fitted by the multinomial logistic regression model, and average growth is the reference group.

[b] Fitted by the generalized estimating equations (GEE).

The main model was adjusted for maternal age, maternal employment status, household income, maternal education, physical activity, pre-pregnancy BMI, parity, fetal sex, season of conception, and average temperature during pregnancy; gestational age was adjusted in the GEE model.

Additionally adjusted model: main model + NO_2, O_3, passive smoking, and dietary energy intake.

Table S8. The pooled association between maternal exposure to PM (**per 5 μg/m³**) and fetal growth in utero with multiple imputed datasets (imputed number=5).

	Main model			Additional model		
	OR/β (95%CI)	*p*	FDR	OR/β (95%CI)	*p*	FDR
**EFW trajectories** [a]						
PM_1						
Slow growth	0.487 (0.224, 1.055)	0.068	0.089	0.351 (0.123, 1.002)	0.051	0.059
Rapid growth	1.570 (1.033, 2.385)	0.035	0.048	1.677 (1.023, 2.750)	0.040	0.050
$PM_{2.5}$						
Slow growth	0.859 (0.544, 1.358)	0.516	0.516	0.758 (0.428, 1.342)	0.342	0.342
Rapid growth	1.451 (1.110, 1.897)	0.007	0.014	1.556 (1.139, 2.126)	0.005	0.008
PM_{10}						
Slow growth	0.862 (0.614, 1.210)	0.390	0.409	0.695 (0.408, 1.182)	0.179	0.188
Rapid growth	1.281 (1.053, 1.557)	0.013	0.021	1.502 (1.144, 1.972)	0.003	0.006
**Z-score** [b]						
PM_1						
BPD	0.078 (0.011, 0.145)	0.023	0.035	0.111 (0.034, 0.188)	0.005	0.008
HC	0.106 (0.040, 0.172)	0.002	0.007	0.135 (0.057, 0.214)	0.001	0.002
AC	0.107 (0.042, 0.171)	0.001	0.007	0.134 (0.058, 0.210)	0.001	0.002
FL	0.057 (-0.011, 0.125)	0.102	0.113	0.081 (-0.001, 0.162)	0.053	0.059
EFW	0.110 (0.045, 0.175)	0.001	0.007	0.140 (0.062, 0.217)	<0.001	0.002
$PM_{2.5}$						
BPD	0.051 (0.013, 0.089)	0.008	0.016	0.070 (0.026, 0.114)	0.002	0.003
HC	0.060 (0.022, 0.099)	0.002	0.007	0.078 (0.033, 0.124)	0.001	0.002
AC	0.058 (0.021, 0.094)	0.002	0.007	0.074 (0.031, 0.117)	0.001	0.002
FL	0.035 (-0.003, 0.074)	0.074	0.092	0.049 (0.002, 0.096)	0.041	0.051
EFW	0.061 (0.024, 0.099)	0.001	0.007	0.079 (0.034, 0.123)	0.001	0.002
PM_{10}						
BPD	0.036 (0.008, 0.065)	0.013	0.021	0.063 (0.025, 0.100)	0.001	0.002
HC	0.042 (0.013, 0.070)	0.004	0.011	0.067 (0.028, 0.105)	0.001	0.002
AC	0.040 (0.012, 0.068)	0.005	0.011	0.061 (0.024, 0.098)	0.001	0.002
FL	0.025 (-0.005, 0.054)	0.098	0.113	0.044 (0.004, 0.084)	0.030	0.043
EFW	0.043 (0.015, 0.072)	0.003	0.009	0.068 (0.030, 0.106)	<0.001	0.002

Abbreviations: PM_1, particulate matter with aerodynamic diameters ≤ 1μm, $PM_{2.5}$, particulate matter with aerodynamic diameters ≤ 2.5μm; PM_{10}, particulate matter with aerodynamic diameters ≤ 10 μm; BPD: biparietal diameter; HC, head circumference; AC, abdominal circumference; FL, femur length; EFW, estimated fetal weight.

[a] Fitted by the multinomial logistic regression model, and average growth is the reference group

[b] Fitted by the generalized estimating equations (GEE).

The main model was adjusted for maternal age, maternal employment status, household income, maternal education, physical activity, pre-pregnancy BMI, parity, fetal sex, season of conception, and average temperature during pregnancy; gestational age was adjusted in the GEE model.

Additionally adjusted model: main model + NO_2, O_3, passive smoking, and dietary energy intake.

Table S9. The longitudinal association between maternal exposure to size-specific PM (**per 5 μg/m^3**) and overgrowth risk (z-score > 1.88) in utero in the GEE model.

	Crude model			Main model			Additional model		
	OR (95% CI)	*p*	FDR	OR (95% CI)	*p*	FDR	OR (95% CI)	*p*	FDR
PM_1									
BPD	1.281 (0.887, 1.850)	0.186	0.235	1.549 (0.985, 2.436)	0.058	0.099	1.822 (0.998, 3.328)	0.051	0.079
HC	1.528 (1.046, 2.232)	0.028	0.085	1.712 (1.090, 2.690)	0.020	0.073	2.073 (1.157, 3.715)	0.014	0.060
AC	1.464 (1.044, 2.052)	0.027	0.085	1.586 (0.982, 2.561)	0.060	0.099	1.993 (1.179, 3.369)	0.010	0.060
FL	0.770 (0.534, 1.112)	0.164	0.235	0.792 (0.555, 1.130)	0.199	0.248	0.783 (0.487, 1.261)	0.315	0.363
EFW	1.268 (0.890, 1.807)	0.188	0.235	1.407 (0.920, 2.150)	0.115	0.157	1.936 (1.086, 3.450)	0.025	0.060
$PM_{2.5}$									
BPD	1.272 (1.026, 1.576)	0.028	0.085	1.394 (1.073, 1.811)	0.013	0.064	1.418 (0.996, 2.020)	0.053	0.079
HC	1.404 (1.116, 1.765)	0.004	0.056	1.495 (1.140, 1.960)	0.004	0.054	1.616 (1.115, 2.342)	0.011	0.060
AC	1.204 (1.001, 1.448)	0.048	0.103	1.322 (1.000, 1.749)	0.050	0.099	1.521 (1.072, 2.157)	0.019	0.060
FL	0.942 (0.744, 1.193)	0.620	0.620	0.948 (0.765, 1.176)	0.627	0.627	0.936 (0.707, 1.239)	0.642	0.642
EFW	1.191 (0.962, 1.474)	0.108	0.180	1.275 (0.993, 1.637)	0.057	0.099	1.471 (1.000, 2.164)	0.050	0.079
PM_{10}									
BPD	1.143 (0.978, 1.335)	0.093	0.175	1.241 (1.026, 1.500)	0.026	0.079	1.288 (0.964, 1.721)	0.087	0.119
HC	1.227 (1.042, 1.444)	0.014	0.085	1.289 (1.064, 1.563)	0.010	0.064	1.412 (1.038, 1.919)	0.028	0.060
AC	1.151 (1.005, 1.318)	0.042	0.103	1.176 (0.964, 1.435)	0.110	0.157	1.382 (1.045, 1.826)	0.023	0.060
FL	0.925 (0.780, 1.098)	0.372	0.398	0.943 (0.799, 1.113)	0.485	0.520	0.920 (0.723, 1.172)	0.501	0.537
EFW	1.087 (0.934, 1.264)	0.281	0.324	1.119 (0.932, 1.345)	0.229	0.264	1.275 (0.939, 1.730)	0.119	0.149

Abbreviations: PM_1, particulate matter with aerodynamic diameters ≤ 1μm; $PM_{2.5}$, particulate matter with aerodynamic diameters ≤ 2.5μm; PM_{10}, particulate matter with aerodynamic diameters ≤ 10 μm.

Crude model was adjusted for nothing;

Main model was adjusted for gestational age, maternal age, maternal employment status, household income, maternal education, parity, fetal sex, season of conception, and average temperature during pregnancy.

Additionally adjusted model: main model + NO_2, O_3, NDVI, passive smoking, and dietary energy intake

Table S10. The associations between fetal growth trajectories in utero and prenatal PM exposure (**per 5 μg/m^3**) with birth weight z-score.

	Birth weight z-score	
	β (95%CI)	*P* value
Fetal growth trajectory		
Average growth	Ref	
Slow growth	-0.71 (-0.99, -0.43)	<0.001
Rapid growth	0.84 (0.67, 1.02)	<0.001
PM exposure		
PM_1	0.05 (-0.07, 0.17)	0.543
$PM_{2.5}$	0.02 (-0.06, 0.11)	0.402
PM_{10}	0.02 (-0.06, 0.10)	0.616

Abbreviations: EFW, estimated fetal weight; PM_1, particulate matter with aerodynamic diameters ≤ 1μm; $PM_{2.5}$, particulate matter with aerodynamic diameters ≤ 2.5μm; PM_{10}, particulate matter with aerodynamic diameters ≤ 10 μm; LGA, large for gestational age.

The logistic regression model was adjusted for maternal age, maternal employment status, household income, maternal education, physical activity, pre-pregnancy BMI, parity, gestational age, fetal sex, season of conception, and average temperature during pregnancy.

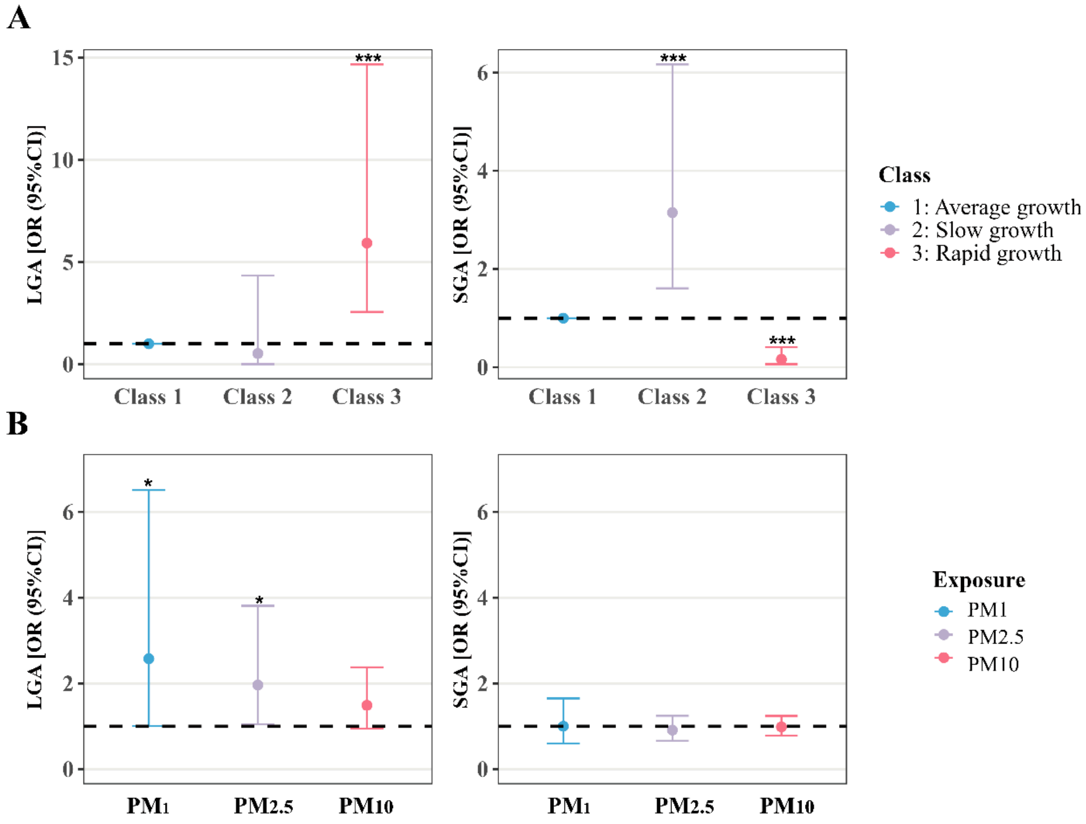

Figure S7. The associations between (A) fetal growth trajectories in utero and (B) prenatal PM exposure (**per 5 μg/m^3**) with LGA and SGA at birth.

Abbreviations: EFW, estimated fetal weight; PM_1, particulate matter with aerodynamic diameters ≤ 1μm, $PM_{2.5}$, particulate matter with aerodynamic diameters ≤ 2.5μm; PM_{10}, particulate matter with aerodynamic diameters ≤ 10 μm; LGA, large for gestational age; SGA, small for gestational age
The regression model was adjusted for maternal age, maternal employment status, household income, maternal education, physical activity, pre-pregnancy BMI, parity, gestational age, fetal sex, season of conception, and average temperature during pregnancy.
*p<0.05, ***p<0.001.

Table S11. Mediation analyses of maternal glucose biomarkers in the association between maternal PM exposure and fetal growth outcomes in the additionally adjusted model.

Mediators/exposure	Total effect	Direct effect	Indirect effect	Proportion [a] mediated
EFW z-score [b] [β (95%CI)]				
FPG				
PM_1	0.146 (0.070, 0.223)	0.136 (0.061, 0.213)	0.010 (0.002, 0.020)	7.2%
$PM_{2.5}$	0.083 (0.040, 0.128)	0.076 (0.033, 0.120)	0.008 (0.003, 0.014)	9.8%
PM_{10}	0.075 (0.037, 0.113)	0.068 (0.03, 0.106)	0.007 (0.003, 0.012)	9.9%
Insulin				
PM_1	0.154 (0.070, 0.24)	0.139 (0.055, 0.225)	0.015 (0.005, 0.028)	10.5%
$PM_{2.5}$	0.086 (0.036, 0.134)	0.083 (0.034, 0.13)	0.003 (-0.002, 0.009)	-
PM_{10}	0.074 (0.030, 0.115)	0.068 (0.025, 0.11)	0.006 (0.001, 0.012)	8.8%
HOMA-IR				
PM_1	0.154 (0.070, 0.240)	0.141 (0.057, 0.226)	0.014 (0.004, 0.028)	9.7%
$PM_{2.5}$	0.086 (0.036, 0.134)	0.082 (0.033, 0.129)	0.004 (-0.001, 0.011)	-
PM_{10}	0.074 (0.030, 0.115)	0.068 (0.024, 0.109)	0.006 (0.002, 0.012)	9.3%
Rapid growth trajectory [c] [OR (95%CI)]				
FPG				
PM_1	1.055 (1.004,1.109)	1.051 (1.002,1.104)	1.003 (0.997,1.011)	-
$PM_{2.5}$	1.068 (1.017,1.124)	1.062 (1.012,1.117)	1.006 (1.000,1.014)	-
PM_{10}	1.085 (1.022,1.153)	1.078 (1.017,1.146)	1.007 (1.000,1.015)	7.6%
Insulin				
PM_1	1.064 (1.009,1.124)	1.056 (1.002,1.114)	1.008 (1.000,1.02)	-
$PM_{2.5}$	1.367 (1.099,1.671)	1.333 (1.081,1.624)	1.025 (0.993,1.067)	-
PM_{10}	1.097 (1.029,1.171)	1.085 (1.019,1.157)	1.011 (1.002,1.023)	11.5%
HOMA-IR				
PM_1	1.065 (1.009,1.125)	1.057 (1.003,1.116)	1.008 (0.999,1.019)	-
$PM_{2.5}$	1.079 (1.023,1.14)	1.073 (1.018,1.131)	1.006 (0.998,1.016)	-
PM_{10}	1.097 (1.029,1.171)	1.085 (1.019,1.156)	1.011 (1.002,1.023)	11.2%

Abbreviations: PM_1, particulate matter with aerodynamic diameters ≤ 1μm, $PM_{2.5}$; particulate matter with aerodynamic diameters ≤ 2.5μm; PM_{10}, particulate matter with aerodynamic diameters ≤ 10 μm; EFW, estimated fetal weight; OR, odds ratio; FPG, fasting plasma glucose; HOMA-IR, homeostatic model assessment for insulin resistance.

[a] Mediation proportions are shown if indirect effects are significant.

[b] Estimated effects for repeated measurements of outcomes associated with a 5-μg/m^3 increase in PM exposure in the generalized estimating equations (GEE)-based mediation analysis.

[c] Estimated effects for the rapid growth trajectory (average trajectory as reference group) in the logistic regression-based mediation analysis; PM levels were scaled by standard deviation.

Proportion mediated was not shown for insignificant indirect effect.

Additionally adjusted model: maternal age, maternal employment status, household income, maternal education, physical activity, pre-pregnancy BMI, parity, fetal sex, season of conception, and average temperature during pregnancy, NO_2, O_3, passive smoking, and dietary energy intake.

Table S12. Mediation analyses of maternal glucose biomarkers in the longitudinal association between PM (per 5 μg/m^3) and z-scores of fetal parameters in utero under a stricter temporal sequence, with PM exposure restricted to the period before glucose assessment and fetal growth measurements restricted to those obtained thereafter.

Outcomes/ mediator	Exposure	Total effect [β (95%CI)]	Direct effect [β (95%CI)]	Indirect effect [β (95%CI)]	Proportion mediated [a]
EFW z-score					
FPG	PM_1	0.079 (0.009, 0.153)	0.070 (0.000, 0.143)	0.009 (0.002, 0.019)	18.2%
	$PM_{2.5}$	0.041 (0.003, 0.08)	0.035 (-0.004, 0.075)	0.006 (0.001, 0.012)	24.7%
	PM_{10}	0.03 (0.002, 0.062)	0.025 (-0.004, 0.057)	0.005 (0.001, 0.010)	23.6%
Insulin	PM_1	0.07 (-0.003, 0.144)	0.055 (-0.021, 0.126)	0.015 (0.005, 0.030)	16.6%
	$PM_{2.5}$	0.033 (-0.007, 0.075)	0.028 (-0.013, 0.069)	0.005 (0.000, 0.012)	36.9%
	PM_{10}	0.022 (-0.01, 0.054)	0.015 (-0.016, 0.047)	0.006 (0.002, 0.012)	33.9%
HOMA-IR	PM_1	0.07 (-0.003, 0.144)	0.056 (-0.021, 0.126)	0.014 (0.004, 0.031)	14.5%
	$PM_{2.5}$	0.033 (-0.007, 0.075)	0.028 (-0.013, 0.068)	0.006 (0.001, 0.013)	37.3%
	PM_{10}	0.022 (-0.01, 0.054)	0.015 (-0.016, 0.047)	0.007 (0.002, 0.013)	35.7%
BPD z-score					
FPG	PM_1	0.037 (-0.041, 0.111)	0.029 (-0.049, 0.105)	0.007 (0.000, 0.018)	18.7%
	$PM_{2.5}$	0.026 (-0.016, 0.067)	0.021 (-0.020, 0.063)	0.005 (0.000, 0.011)	10.1%
	PM_{10}	0.018 (-0.016, 0.051)	0.014 (-0.020, 0.047)	0.004 (0.000, 0.009)	22.6%
Insulin	PM_1	0.034 (-0.049, 0.114)	0.028 (-0.056, 0.11)	0.006 (-0.002, 0.015)	-
	$PM_{2.5}$	0.022 (-0.023, 0.067)	0.019 (-0.025, 0.066)	0.002 (-0.001, 0.006)	-
	PM_{10}	0.009 (-0.026, 0.045)	0.007 (-0.029, 0.043)	0.003 (-0.001, 0.007)	-
HOMA-IR	PM_1	0.034 (-0.049, 0.114)	0.027 (-0.055, 0.109)	0.006 (0.000, 0.016)	-
	$PM_{2.5}$	0.022 (-0.023, 0.067)	0.019 (-0.026, 0.065)	0.003 (0.000, 0.007)	-
	PM_{10}	0.009 (-0.026, 0.045)	0.006 (-0.029, 0.042)	0.003 (0.000, 0.007)	-
HC z-score					
FPG	PM_1	0.08 (0.008, 0.154)	0.074 (0.004, 0.148)	0.005 (-0.001, 0.015)	-
	$PM_{2.5}$	0.047 (0.008, 0.088)	0.044 (0.005, 0.084)	0.003 (-0.001, 0.009)	-
	PM_{10}	0.033 (0.003, 0.065)	0.030 (0.000, 0.062)	0.003 (0.000, 0.007)	-
Insulin	PM_1	0.071 (-0.007, 0.148)	0.062 (-0.017, 0.139)	0.009 (0.001, 0.019)	12.2%
	$PM_{2.5}$	0.04 (-0.002, 0.082)	0.037 (-0.005, 0.079)	0.003 (0.000, 0.008)	6.9%
	PM_{10}	0.023 (-0.009, 0.057)	0.019 (-0.013, 0.053)	0.004 (0.001, 0.008)	24.9%
HOMA-IR	PM_1	0.071 (-0.007, 0.148)	0.063 (-0.015, 0.139)	0.008 (0.001, 0.020)	11.5%
	$PM_{2.5}$	0.040 (-0.002, 0.082)	0.037 (-0.006, 0.079)	0.003 (0.000, 0.008)	8.2%
	PM_{10}	0.023 (-0.009, 0.057)	0.019 (-0.013, 0.054)	0.004 (0.001, 0.009)	25.5%
AC z-score					
FPG	PM_1	0.082 (0.01, 0.157)	0.074 (0.003, 0.149)	0.007 (0.001, 0.016)	14.2%
	$PM_{2.5}$	0.041 (0.002, 0.082)	0.036 (-0.002, 0.077)	0.005 (0.000, 0.010)	21.0%
	PM_{10}	0.031 (0.002, 0.064)	0.027 (-0.003, 0.06)	0.004 (0.000, 0.008)	14.5%
Insulin	PM_1	0.082 (0.007, 0.16)	0.066 (-0.012, 0.141)	0.017 (0.005, 0.033)	28.6%

	$PM_{2.5}$	0.04 (-0.001, 0.082)	0.034 (-0.008, 0.076)	0.006 (0.000, 0.013)	11.4%
	PM_{10}	0.028 (-0.005, 0.061)	0.021 (-0.012, 0.054)	0.007 (0.003, 0.013)	18.4%
HOMA-IR	PM_1	0.082 (0.007, 0.16)	0.067 (-0.012, 0.144)	0.015 (0.005, 0.033)	28.9%
	$PM_{2.5}$	0.04 (-0.001, 0.082)	0.034 (-0.008, 0.076)	0.006 (0.001, 0.014)	9.1%
	PM_{10}	0.028 (-0.005, 0.061)	0.021 (-0.013, 0.054)	0.007 (0.003, 0.014)	18.7%

Abbreviations: PM_1, particulate matter with aerodynamic diameters ≤ 1μm; $PM_{2.5}$, particulate matter with aerodynamic diameters ≤ 2.5μm; PM_{10}, particulate matter with aerodynamic diameters ≤ 10 μm; FPG, fasting plasma glucose; HOMA-IR, homeostatic model assessment for insulin resistance; BPD: biparietal diameter; HC, head circumference; AC, abdominal circumference; EFW, estimated fetal weight.

[a] Mediation proportions were shown if indirect effects are significant.

The generalized estimating equations (GEE)-based mediation model was adjusted for gestational age, maternal age, maternal employment status, household income, maternal education, physical activity, pre-pregnancy BMI, parity, fetal sex, season of conception, and average temperature during pregnancy. Maternal employment status, physical activity, and pre-pregnancy BMI had missing data for 5.0% (n=31), 2.6%(n=16), and 3.2% (n=20), which were handled with the Multivariate Imputation by Chained Equations algorithm.
